# Shape-Memory Materials via Electrospinning: A Review

**DOI:** 10.3390/polym14050995

**Published:** 2022-02-28

**Authors:** Valentina Salaris, Adrián Leonés, Daniel Lopez, José Maria Kenny, Laura Peponi

**Affiliations:** 1 Instituto de Ciencia y Tecnología de Polímeros (ICTP-CSIC), C/Juan de la Cierva 3, 28006 Madrid, Spain; v.salaris@ictp.csic.es (V.S.); aleones@ictp.csic.es (A.L.); daniel.l.g@csic.es (D.L.); 2 Interdisciplinary Platform for “Sustainable Plastics towards a Circular Economy” (SUSPLAST—CSIC), 28006 Madrid, Spain; 3 STM Group Strada di Pentima 4, Civil and Environmental Engineering Department and UDR INSTM, University of Perugia, 05100 Terni, Italy; jose.kenny@unipg.it

**Keywords:** electrospinning, nanocomposites, shape memory, biopolymers, smart materials, electrospun fibers, biomedical applications

## Abstract

This review aims to point out the importance of the synergic effects of two relevant and appealing polymeric issues: electrospun fibers and shape-memory properties. The attention is focused specifically on the design and processing of electrospun polymeric fibers with shape-memory capabilities and their potential application fields. It is shown that this field needs to be explored more from both scientific and industrial points of view; however, very promising results have been obtained up to now in the biomedical field and also as sensors and actuators and in electronics.

## 1. Introduction

**Figure 1:**
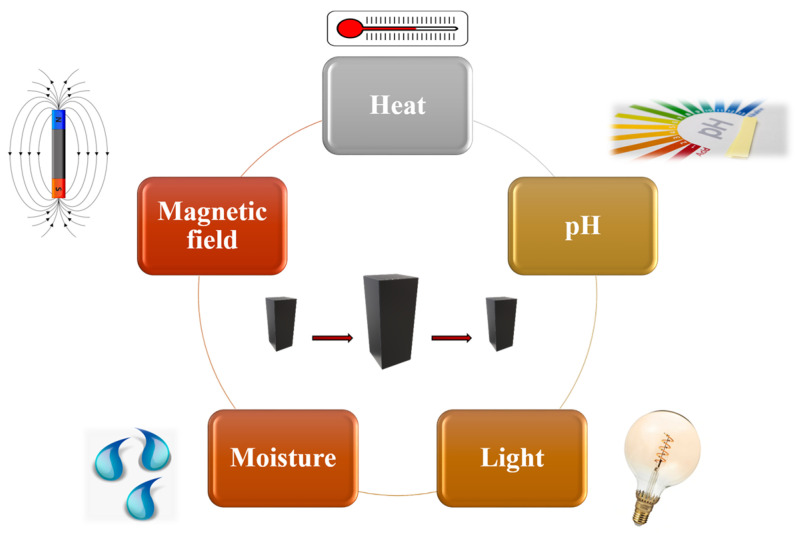
Stimuli-responsive shape-memory polymers.

Shape-memory polymers (SMPs) are an emerging class of smart materials that have been widely studied since the 1980s and are capable of recovering their initial shape from a temporary shape through the application of external stimuli: light, temperature, pH, magnetism, electricity, or moisture [1,2,3,4], as schematically represented in Figure 1. Furthermore, such materials are able to memorize one temporary shape (two-way SMPs) or several shapes (multi-shape SMPs) [5].

However, the shape-memory behavior is not an intrinsic characteristic but can be achieved by designing the molecular structure of the polymer and by setting both the programming and the recovering stages that are generally required in order to fix the temporary shape and to recover the original shape of the polymer, respectively [6]. In particular, an SMP requires switching domains, constituted by side chains or chain fragments in which the interactions between such segments stabilize the temporary shape [6] and net-points (network), formed by covalent bonds (chemical cross-linking) or by intermolecular interactions, molecule entanglement, crystalline phases, or interpenetrated networks. These chemical or physical network points determine the original shape while the switching segments, which are the part of the material that responds to the external stimulus, fix the temporary shape [7]. 

**Figure 2:**
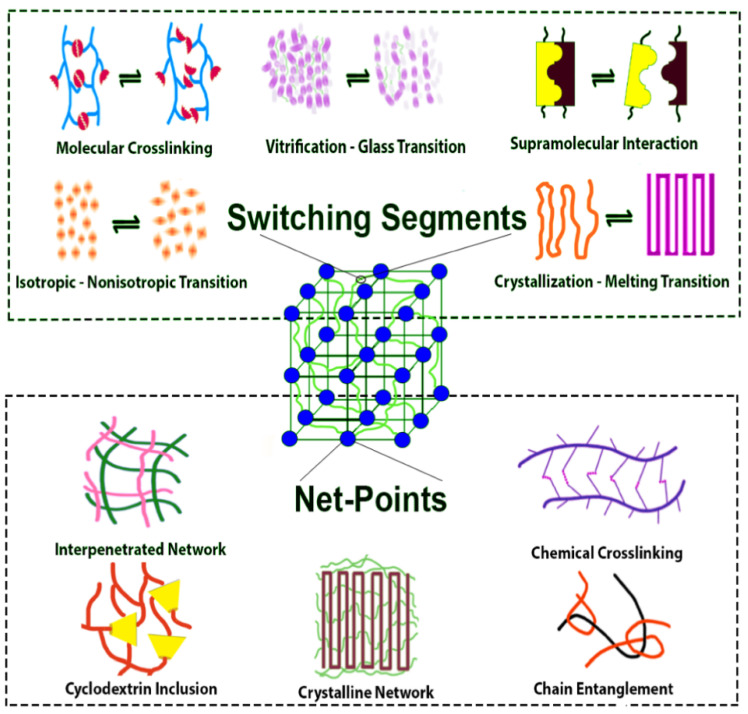
Shape-memory polymers architecture and possible transitions [9].

Different transitions may be used to establish the temporary shape, including crystallization/melting transitions, vitrification/glass transitions, liquid crystal anisotropic/isotropic transitions, reversible molecule cross-linking, and supramolecular association/disassociation [8] (Figure 2). 

**Figure 3:**
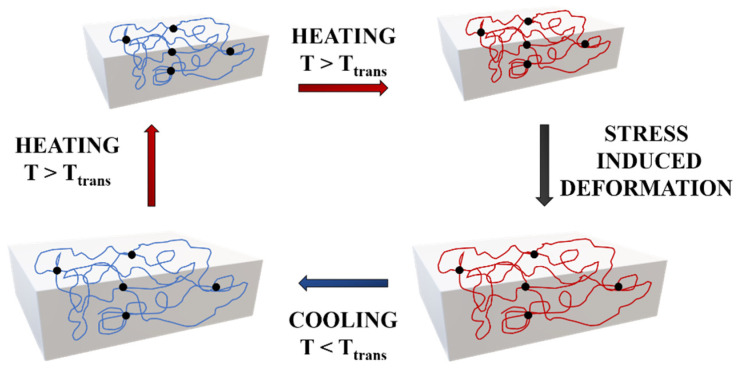
Mechanism of thermally induced shape-memory polymers.

Actually, the most-used SMPs are thermally-induced shape-memory polymers. Their typical mechanism involves heating above a specific temperature, called the transition temperature (T_trans_), which can initiate some of the transformations mentioned above. One of the most-used temperatures is the glass transition temperature (T_g_), at which the materials turn from a rigid state to a soft state. This phenomenon occurs due to the increased mobility of the polymeric chains when heating the sample above T_g_, and therefore, when an external force is applied, the material can be deformed into a desired temporary shape that corresponds to a change in the molecular conformation and entropy. When cooling below T_g_ and by gradually removing the external force, the temporary shape can be fixed through freezing the molecular chains and the applied force is stored as entropic energy inside the system. Upon reheating above the T_g,_ the polymeric chains acquire more mobility, allowing the release of the entropic energy, which is the driving force of the process, and the material is driven back to its entropically favorite state that corresponds to the initial shape [10] (Figure 3). 

The performance of an SMP can be evaluated through specific parameters: the *strain fixity ratio R_f_* and the *strain recovery ratio R_r_*, which refer to the capability of the material to fix the mechanical deformation and to memorize its permanent and original shapes, respectively, and can be easily estimated by the following equations [11,12]:

$$R_{f\;}\left(N\right)=\frac{\varepsilon_{\;u}\;\left(N\right)}{\varepsilon_{\;m}}\times 100\%$$

$$R_{r}\left(N\right)=\frac{\varepsilon_{\;m}-\varepsilon_{p\;\left(N\right)}}{\;\varepsilon_{\;m}-\varepsilon_{p\;\left(N-1\right)}}\times 100\%$$

where ε_m_ is the maximum strain after cooling and before releasing the stress. When cooling at T < T_trans_ and the stress is removed, the sample strain corresponds to a fixed strain, ε_u_. By reheating, the sample returns to its original shape, leading to a recovered strain, ε*_p_*.

**Figure 4:**
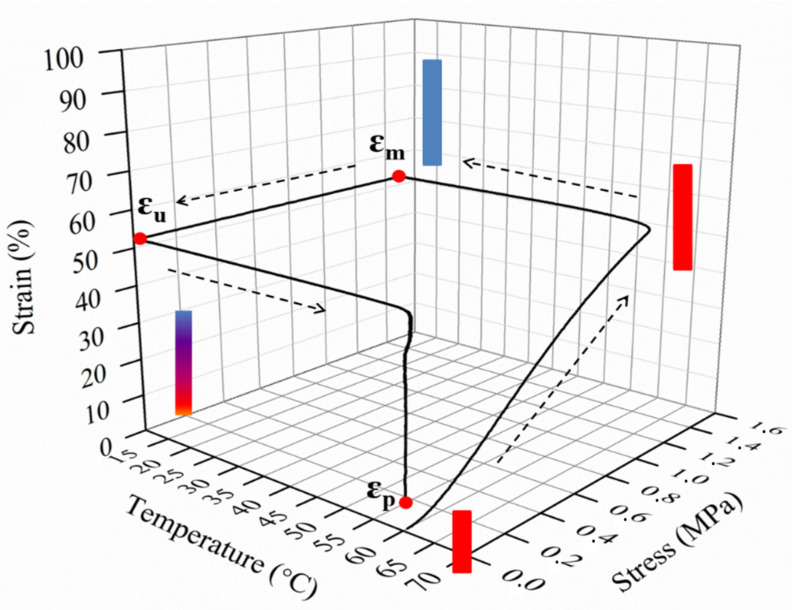
Schematic diagram of a 3D thermo-mechanical cycle and main parameters.

The shape-memory properties of a specific material can be studied through thermo-mechanical cycles, as reported in Figure 4. 

Many researchers have focused their attention on SMPs due to the various advantages compared with shape-memory alloys: low density and relatively low cost, high recoverable strain (up more than 200% for most of the materials) [11], biocompatibility, and biodegradability. In addition, the transition temperature of SMPs with respect to SMA can be easily tailored. SMA presents a well-defined transition temperature, while for polymers it can be tailored through different approaches. That is, by the specific molecular design of the material, by blending, copolymerizing, as well as reinforcing the polymers, or by inducing crystallization in the material and therefore varying the stiffness of the polymer and, indeed, its transition temperature [13]. Furthermore, SMPs can be widely applied in many areas, such as in biomedical devices [14], aerospace [15], packaging [16], sensors [17], bionics engineering [18], electronic engineering [19], nano-optoelectronic applications [20], etc. 

Among these materials, shape-memory fibers have been studied since the fibers possess particular intrinsic features such as a high axial ratio, and compared with shape-memory polymer bulk, shape-memory fibers exhibit excellent mechanical properties because of their molecular orientation. Many polymers have been used for electrospinning, such as poly(vinyl pyrrolidone) (PVP), poly(vinyl acetate) (PVAc), polystyrene (PS), poly(vinyl alcohol) (PVA), poly(ethylene oxide) (PEO), polyacrylonitrile (PAN), polyethersulfone (PES), poly(ε-caprolactone) (PCL), and poly(lactic acid) (PLA) [21,22,23,24]. Several spinning techniques are adopted to produce conventional fibers: wet spinning, dry spinning, reaction spinning, gel spinning; however, electrospinning has received great attention since it has been proved to be a simple and promising technique [25,26] for processing polymeric fibers. 

**Figure 5:**
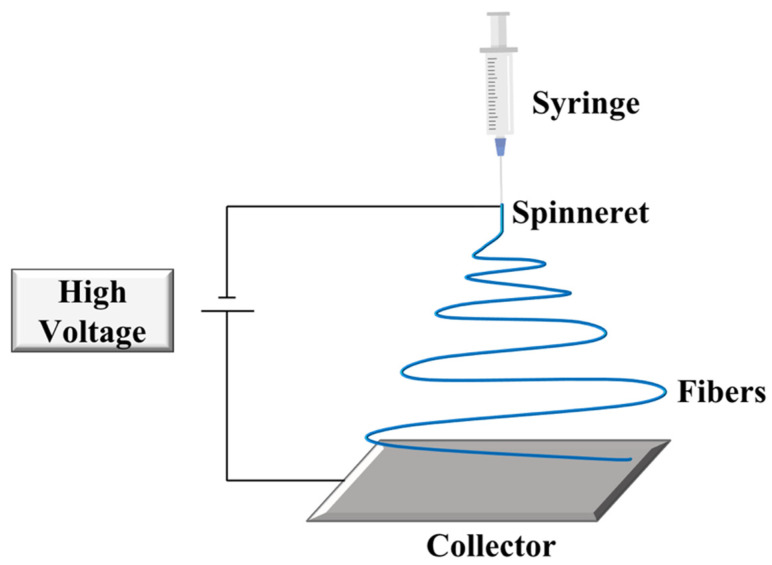
Schematic representation of an electrospinning setup.

Electrospinning was developed by Formhals in 1934 [27] and produces a mat of fibers by applying an electrostatically driven jet of polymer solution or polymer melt. A solution electrospinning system consists of three major constituents: a high voltage supply, a spinneret, and a collecting plate. A high voltage power supply is required to obtain an electrically charged jet of the solution. Indeed, the electric field between the capillary tip and a collector induces charges on the surface of the solution or melt [28]. By increasing the electric field, the hemispherical shape of the pendant drop at the end of the capillary tip is charged and assumes a conical shape, known as a “Taylor Cone” [29]. When the electric field is induced above a critical value, the surface tension is overcome by the repulsive electrical forces, the charged jet of the solution is ejected from the tip, and the fibers are collected [30] (Figure 5). This method requires the application of high electric voltages, in a range between 10 and 30 kV, and large distances (15–30 cm) between the electrodes. Additionally, the fiber formation strongly depends on several variables such as surface tension, viscosity, the concentration of the solution, solvent, etc. [31]. 

Electrospinning is a technique that produces polymeric fibers of nanometer to micrometer sizes in diameter [32] and has been widely used to produce fibers with diameters ranging from 50 to 500 nm [33]. Indeed, if the fiber diameter is reduced from micrometers to nanometers, certain properties can be significantly modified; in particular, small pore size and high specific surface area [34], features that can be exploited in soldier protective clothing, filtration, membranes, reinforcing fibers in composite materials, optical and electronic applications, drug delivery, cosmetics, supports for enzymes or catalysts, scaffolds for tissue engineering, H_2_ production, environmental protection, antibacterial properties, etc. [35,36,37,38,39,40,41,42].

**Figure 6:**
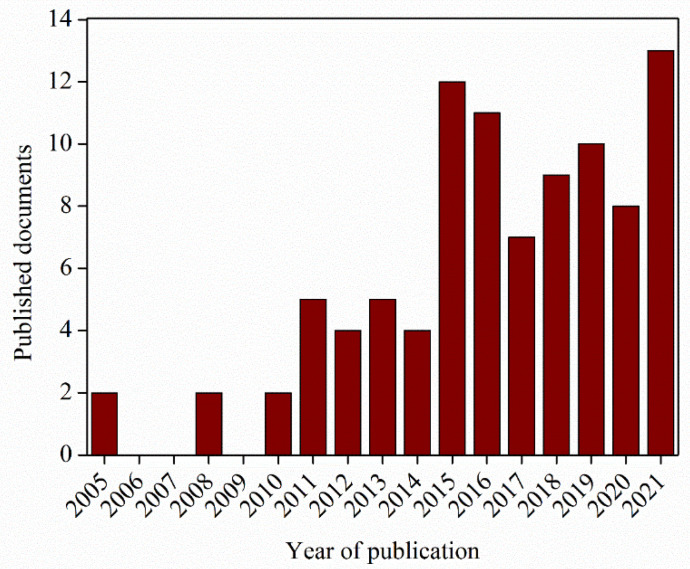
Diagram of the number of documents regarding shape-memory fibers per year (Scopus Source).

In the last decades, researchers have tried to combine the features of polymer electrospun nanofibers with SM behavior, developing materials with improved properties. However, this is still considered a new, challenging research topic, since there are not many documents regarding shape-memory fibers (Figure 6): fewer than 15 scientific papers/year in the last 7 years. The first publications were mostly focused on the study of the morphology and optimization of the various parameters in order to fabricate fibers with a lack of defects and controlled diameters, whereas successive publications were mainly centered on the development of fibers for specific applications, in particular in the biomedical field, such as tissue engineering, wound dressing, drug delivery, and others such as catalysis, sensors, robotics, aerospace technologies, and more. All these works are reported and described in the present review.

## 2. Study of SMP Structure and Properties

**Figure 7:**
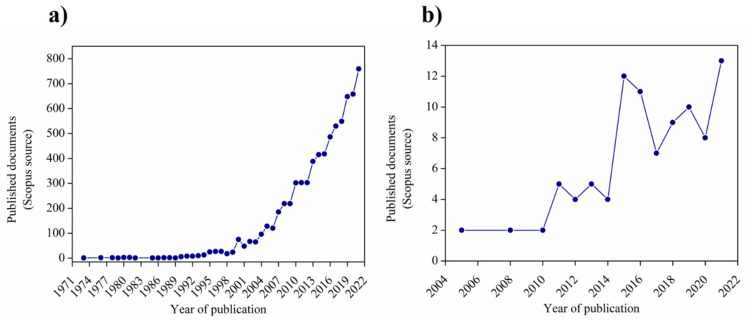
Number of published scientific papers looking for keywords: (**a**) “shape memory” + “polymers”; (**b**) “shape memory” + “polymers” + “electrospinning”.

Shape-memory polymers (SMPs) have received great attention for their potential applications in many fields. Indeed, the number of published papers has increased in recent years. Figure 7a reports the schematic trend of scientific articles from the Scopus database, searching with keywords “shape memory” and “polymer”, in which we can see the exponential growth that started 20 years ago. In Figure 7b, for comparison, we report the same schematic trend using, as keywords, “shape memory”, “polymer”, and “electrospinning”. In particular, shape-memory fibers have been widely studied for designing functional materials for human beings, finding applications in clothing fabrication [25], the biomedical field [14], robotics [15], catalysis, and air and water filters [7,43].

Therefore, in recent years, new polymer fibers have been developed and significant advances have been carried out in the production of high-performance fibers, elastic fibers, and nanofibers by using the electrospinning method [25]. The first works were mainly focused on the study of the structure and on controlling the diameter of fibers and the properties of these materials. Boland et al. [44] characterized the mechanical properties of polydioxanone (PDS) fibers, which are generally extruded into a filament to use as sutures. They noticed that, as the solution concentration increased, the diameter of the fibers increased linearly, and as the diameter of the fibers increased, the values of the Young modulus, peak stress, and strain at failure increased as well. However, mechanical tests revealed the anisotropic properties of the oriented mat. 

One of the most studied shape-memory polymers is the class of polyurethanes (PUs), introduced by Mitsubishi in 1988 [45]. Shape-memory PU consists of hard and soft segments, where hard segments, which can be formed through hydrogen bonds and crystallization, are built from diisocyanates and act as physical crosslinks, while soft segments are polyols and are responsible for the shape-memory effect [46,47]. Cha, et al. [48] prepared shape-memory PU block copolymers with various hard-segments ratios, and the mechanical analysis revealed that the tensile strength increased as the hard-segment concentration increased, owing to the effect of the rigid diphenyl methylene moiety combined with hydrogen bonding and the dipole–dipole interaction. In addition, the tensile strength improved as the viscosity increased; indeed, at low viscosity, the woven no-woven electrospun PU assumed a beaded-on-fibers structure, where the beads act as defects. Lastly, they obtained more than 80% of shape recovery with hard segment concentration of 40 and 50%. 

Peponi et al. studied a shape-memory poly(ester-urethane) based on triblock copolymers based on poly(L-lactic acid) (PLLA) and PCL [11,49]. They obtained excellent shape-memory responses at 40 °C in terms of both *R_r_* and *R_f_* [11]. They also used this polymer as a matrix for nanocomposites reinforced with cellulose nanocrystals, maintaining their shape-memory behavior at 35 °C [49]. However, they did not obtain electrospun fibers without defects of this material [50]. 

In order to combine the properties of SMPs with nanofibers, Zhuo et al. [34] tried to develop shape-memory polyurethane nanofibers by electrospinning, noting that the diameters of the fibers decreased by increasing the applied voltage and by decreasing the concentration: nanofibers with a diameter of 50–100 nm were electrospun from the 3.0 wt.% solution, a diameter of 200–400 nm from the 7.0 wt.% solution, and a diameter of 500–700 nm from 12.0 wt.% solution. Cyclic tensile tests were executed to investigate the SM performances: the membrane showed an *R_r_* value of 98% and an *R_f_* value of 80% after several cyclic times.

In general, SMPU possesses unique properties such as low production cost, low density, flexibility, good processability, and high deformation, but still has some disadvantages such as poor surface wettability, low shape-memory effect, and mechanical strength, thus additives or reinforcements are often incorporated in order to improve these features [51]. Rana et al. [52] prepared core-shell nanofibers by co-electrospinning made of PU core and PU composite shells reinforced with multi-walled carbon nanotubes (MWNTs). Compared with pure PU nanofiber webs, the incorporation of MWNTs lead to a better shape-memory behavior (especially with 1.0 wt% MWTNs content) due to the high thermal conductivity coefficient of MWNTs [53] and the higher internal stress stored by PU/MWNTs, owing to the strong interaction between matrix and reinforcement. 

Comparing carbon nanotubes and graphene, the graphene oxide (GO), which possesses several oxygen-containing groups [54] and weakens the van der Waals interactions between the GO sheets, is easier to disperse into a polymer matrix. Tan et al. [51] fabricated a series of GO-reinforced SMPU nanofibers and the SM behavior was evaluated. In particular, they reported that all the samples showed an *R_f_* and *R_r_* above 90% (92.1% for the sample with the highest content in GO); instead, the neat sample revealed an *R_f_* value of 89.2%. These results were explained by the presence of GO as reinforcement with a large specific surface area and a very good dispersion into the matrix, and therefore, it improved the modulus, which is advantageous for a better shape fixation [55].

Subsequent studies have been based on the production of polymer-based scaffolds. In 2015, Zhang et al. [56] developed a fiber scaffold constituted by hollow microfibers composed of a degradable shape-memory copolyetheresterurethane (PDC). PDC was constituted by crystallizable oligo(p-dioxanone) (OPDO) hard and oligo(ε-caprolactone) (OCL) switching segments. The hollow microfibers were fabricated by coaxial electrospinning where poly(ethylene glycol) (PEG) was used as a “sacrificial” core and was removed afterward with water. The microfibers were produced with the same average diameter of 1.4 μm but with different degrees of hollowness—that is 0, 13, and 33%—and they observed that the shape-memory performance improved (shape recovery in particular) with the increase in fiber hollowness.

Another interesting study was carried out by Alhazov et al. [57], by developing an electrospun thermoplastic polyurethane (TPU) and studying its thermomechanical behavior and shape-memory capabilities. In previous work, they observed that upon heating at ~70 °C, the nanofibers were subjected to contraction, while the TPU cast film was subjected to expansion [58]. Therefore, the thermomechanical and SM properties of the nanofibers were studied and compared to those of the TPU cast films, which was submitted to a typical thermomechanical cycle. During electrospinning, the fibers underwent deformation followed by a contraction due to the decreased molecular mobility caused by the rapid solvent evaporation, thus the electrospinning process was identified to be like the programming cycle, while the contraction effect was identified as the recovery phase. Basically, the contraction of electrospun TPU materials could be considered an electrospinning-induced shape-memory effect. 

Other studies based on PU were performed by Budun et al. [59], by fabricating shape-memory block co-polymer PU fibers with four different solution concentrations (5, 10, 15, and 20 wt.%) and three different voltages (30, 35, and 38.9 kV) and shape-memory properties were evaluated: all calculated *R_f_* were above 80% and all *R_r_* around 100%.

**Figure 8:**
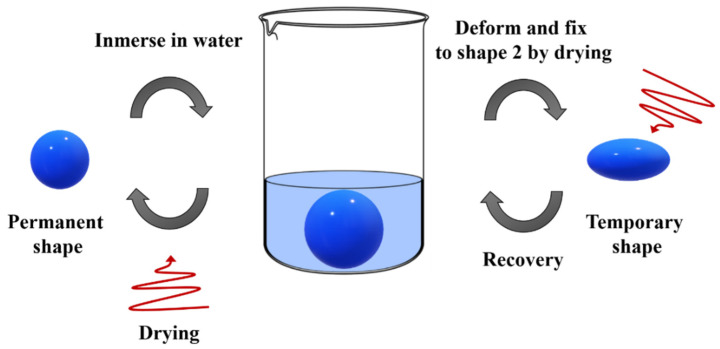
Mechanism of water-responsive shape recovery.

Moreover, some researchers have been focusing their attention on the processing of copolymers to achieve porous structures. Especially, Ang et al. [60] fabricated water-responsive urethane-based shape-memory polymers constituted by PEG, PCL, and poly(dimethylsiloxane) (PDMS). In particular, a water-responsive SMP system should consist of hydrophilic parts as switching segments and hydrophobic parts as hard segments. In contact with water, hydrophilic components swell, leading to a disruption of the crystalline structure, and deformation is achieved (programming). Therefore, the temporary shape can be set by drying, and the crystalline structure is reinstated [61] (Figure 8). 

The electrospinning technique has been used also to produce porous structures in order to increase the content of water incorporated, and indeed their surface area [62,63]. The PEG crystals constitute the hydrophilic component, which is able to interact with water, whereas PCL and urethane linkages constitute the hydrophobic component, holding the permanent structure. The corresponding electrospun mat exhibited a faster recovery than the bulk SMP due to the higher surface area in contact with water. 

**Figure 9:**
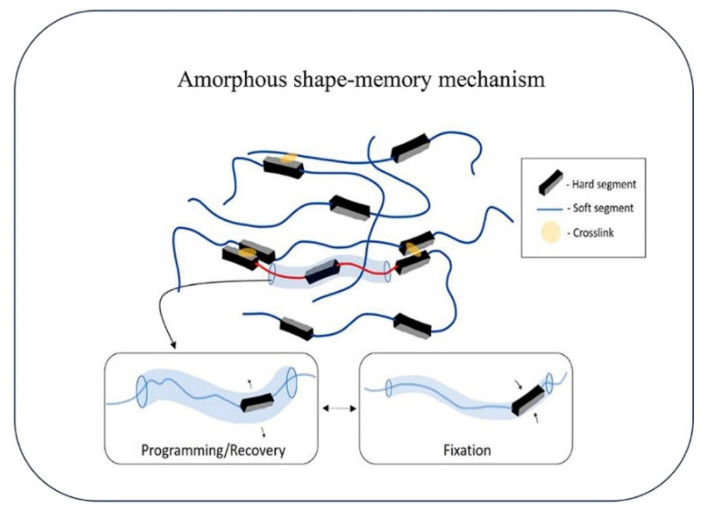
Shape-memory amorphous mechanism [66].

Induced, crystallization is quite an innovative manner in which to obtain shape-memory materials. Sessini et al. [64] proposed inducing crystallization in an ethylene-vinyl acetate (EVA)–thermoplastic starch (TPS) system in order to obtain both thermally and humidity-activated shape-memory materials. At the same time, the induced crystallization in the PCL domains of PU was used by Castillo et al. [65] to obtain shape-memory materials with potential applications as vascular grafts. In this regard, one of the most recent works based on polyurethane was performed by Nissenbaum et al. [66], by combining polycaprolactone-diol (PCL-diol)/2,4,2,6-toluene diisocyante (TDI) and ethylene glycol as a chain extender to obtain SMPU nanofibers by electrospinning. Moreover, they studied strain-driven crystallization, which is the mechanism that regulates the SMPU fixation state in semi-crystalline SMPUs [67]. In fact, they reported that when the polymer chains are deformed under strain, the co-alignment keeps the chains close and induces crystallization [68], while when cooling below the transition temperature, usually the melting temperature (T_m_), the strain-induced crystallization fixes the temporary shape. However, after heating, the SMPU becomes amorphous and maintains this state for more than 60 h, delaying its crystallization. This effect allows for a complete shape-memory cycle to be carried out without using the effect of crystallization, and the authors observed the excellent shape-memory performance of the SMPU in the amorphous state even without the help of the crystallization. Therefore, they suggested that the fixation and recovery shape mechanisms are caused by the inhibition of the chain mobility caused by interlocking the hard segments when decreasing the temperature below the transition temperature, and subsequent heating allows free chain movement and the recovery of the initial shape (Figure 9). The electrospun SMPU fibers they studied showed a value of ~100 and 97% for strain recovery and fixity ratios, respectively.

Chen et al. [69] designed a shape-memory film by using as a precursor a γ-aminopropyltriethoxysilane (APS) end-capped polyurethane oligomer (ASPU) that was subsequently hydrolyzed and condensed through the ethoxysilane groups, and finally crosslinked through a sol-gel process after electrospinning. The shape-memory performance was evaluated by carrying out three cycles in the temperature range of 0 and 60 °C and by applying a strain of 150% obtaining *R_f_* and *R_r_* values of 99.1 ± 0.4% and 81.3 ± 0.8%, respectively. 

In addition to the class of polyurethanes, PCL has been extensively studied. As one of the biodegradable polyesters and for its great biocompatibility, it can be used in the biomedical field and packaging applications [70,71]. Nevertheless, the applications of these polymers are limited because of their deficient physical properties; indeed, polymer/inorganic layered material nanocomposites or the copolymers of PCL have been investigated. Matsumoto et al. [72] explored the shape-memory behavior of the electrospun woven no-woven fabrics of a multiblock copolymer named PDLCL, constituted by poly(x-pentadecalactone) (PPDL) as hard segments and PCL as soft and switching segments. They calculated the shape-memory capability at 40 and 60 °C and by using two different methods: method (A), where a maximum strain of ε_m_ = 25% was applied for each cycle, while in method (B), ε_m_ was subsequently increased from 25 to 50%, and *R_r_* values between 89% and 95% and *R_f_* values of 82–83% after the second cycle were obtained when small deformations at clinically relevant temperatures were applied. 

Luo et al. [73] incorporated thermoplastic fibers of a low T_m_ semicrystalline polymer into a T_g_-based SMP matrix to develop triple-shape polymeric composites (TSPCs), which are a class of smart materials capable of fixing two temporary shapes besides their permanent shape [74]. PCL and epoxy were chosen for this purpose. In fact, epoxies are very interesting materials frequently used due to properties such as chemical resistance, high stiffness, good adhesion, and versatility [75]. In particular, they reported that the programming of the two temporary forms was set at two temperatures: the melting temperature of the PCL crystals (T_m_ of 40 °C) and the T_g_ of EP (T_g_ of 80 °C), and dual and triple SM performances were investigated, revealing *R_f_* and *R_r_* values close to 100% for the former case, whereas *R_f_* and *R_r_* values for the second shape were lower than the values of the first one. 

A similar approach was applied by Fejos et al. [76] by developing a triple shape-memory polymer (TSMPs) of epoxy (EP)/PCL with diverse structures. PCL was present as a nanoweb, electrospun with and without graphene (graphene was incorporated as reinforcement and as a spacer to increase the impregnation with EP) and in situ formed with a co-continuous phase structure. Both the T_g_ of the EP and the T_m_ of PCL were used as transition temperatures to fix the two temporary shapes. From the shape-memory performance, it was observed that EP/PCL nanoweb with graphene showed worse SM properties than the unreinforced material, and additionally, EP/PCL with a co-continuous structure revealed the best properties with *R_f_*_1_, *R_f_*_2_, *R_r_*_1_, and *R_r_*_2_ values of 81–82%, 94–95%, 94%, and 85%, respectively.

Yao et al. [77] elaborated a thermosetting/thermoplastic system comprised of EP resin and PCL, where PCL microfibers were used as plasticizers and as hard segments to set the temporary shape. The mechanism of the shape-memory effect was attributed to the structure acquired (structural design) by the thermoplastic network inside the thermosetting matrix rather than the molecular structure itself. The recovery of the initial shape was triggered by placing the spiral strip (temporary shape) in hot water (70 °C) and an R_r_ around 97% was observed over the 20 cycles, and an *R_f_* around 95.5%.

Birjandi et al. [78] reported in 2016 the incorporation of PCL electrospun fibers into a shape-memory EP matrix in order to develop a shape memory-based self-healing coating. In particular, shape memory can be used to fabricate self-healing (SH) polymers that are able to close and repair cracks when thermally activated [79]. 

**Figure 10:**
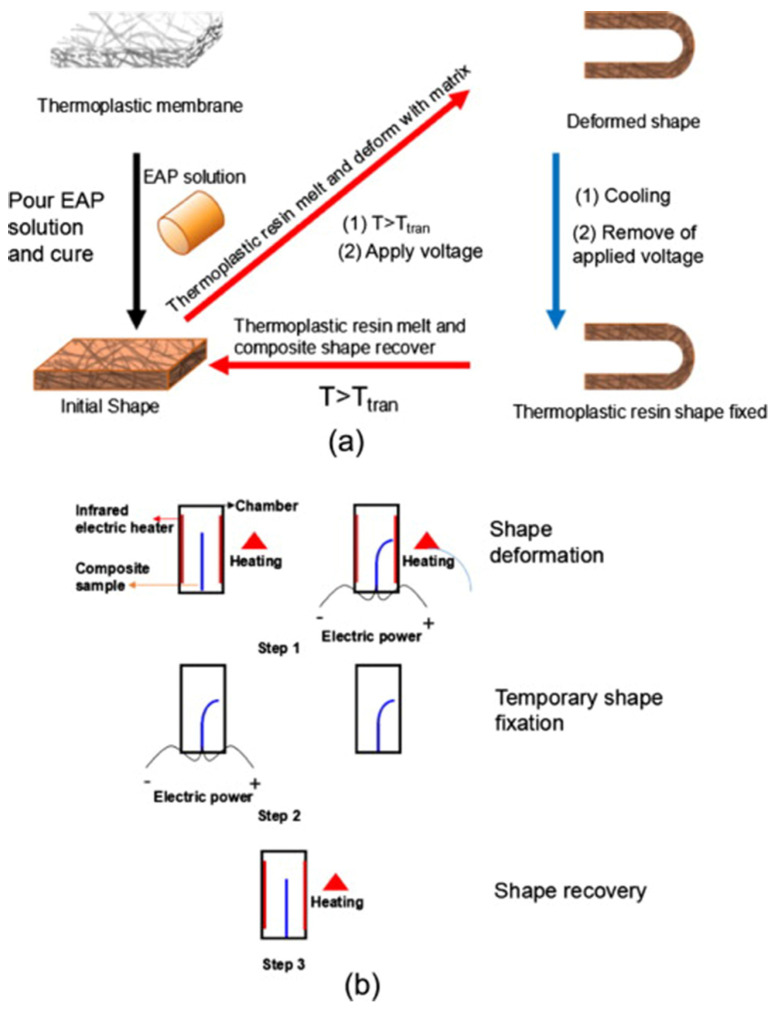
(**a**) Representation of composite fabrication and shape-memory cycles; (**b**) schematic experimental setup for two-way SMPs [80].

In 2016, Yao et al. [80] fabricated a two-way shape-memory composite of electroactive (EAP)/PCL, where PCL was used as a hard segment and the EAP matrix as a soft segment. EAP are generally subjected to deformation when exposed to an electric field. Furthermore, their biocompatibility and similarity with muscles make them suitable for applications as artificial muscles in the biomedical field [81]. This composite showed the ability to change its shape without the pre-programming step by increasing the temperature above the T_m_ of PCL, thus the PCL crystal melts, meanwhile a voltage is applied on the EAP, causing the deformation. Once the material assumed the desired shape, the heat was removed while the voltage was still applied to keep the shape, and when the temperature reached values below the T_m_, the temporary shape was fixed and the voltage was removed. The initial shape was recovered by heating the composite (Figure 10). In this case, the shape-memory behavior was evaluated, revealing an *R_r_* value of 99 ± 0.3%.

In 2016, Marlettini et al. [82] developed an electrospun mat constituted by α,ω-triethoxysilane-terminated PCL to produce beads-free fibers by using electrospinning and sol-gel reaction in order to obtain a material with a high crosslinking degree. The evaluation of the shape-memory properties revealed high values of *R_f_* (100%) and *R_r_* (98%) and the independence of these properties from the content of crosslinking. 

In 2017, Iregui et al. [83] obtained an electrospun mat constituted by a blend of the diglycidyl ether of bisphenol A (DGEBA) and PCL that was subsequently reticulated by exposition to UV radiation, avoiding the melting of the PCL crystals. Iodonium salt was used as a photo-initiator, which also improved the spinnability of the solution, leading to the formation of fibers without defects. The produced mats exhibited high shape-memory properties through several cycles, with an *R_f_* of 95–99% and an *R_r_* of 88–100%, respectively. Moreover, on the basis of this work, they evaluated the effect of the solution concentration, photo-initiator concentration, and blend composition (PCL/epoxy ratio) on the spinnability and shape-memory properties [84]. They noticed that the concentration of PCL did not affect the fixation process, which is regulated by the melting and re-crystallization of PCL: in fact, the degree of crystallinity (ranging from 23 to 40%) was high enough to maintain the temporary shape. However, the concentration of PCL affected the morphology of the fibers and their recovery capability. Indeed, when increasing the solution concentration, the fiber diameter increased. Apparently, a higher diameter of fibers yields better performance in recovering the permanent shape. Moreover, the epoxy and/or the photo-initiator amount showed no effects on the shape-memory behavior, probably due to the high curing degree, as revealed by Fourier Transform Infrared Spectroscopy (FTIR) analysis. In any case, all samples showed excellent shape-memory behavior with *R_f_* and *R_r_* values between 95% and 100%.

Furthermore, many studies were focused on the processing of multiphase shape-memory materials. For instance, Buffington et al. [85] designed quadruple shape-memory composites, which are materials that can recover their original shape from three formerly programmed shapes [86], constituted by an electrospun mat of PCL, epoxy monomer, and poly(methyl methacrylate) (PMMA), and with three distinct transition temperatures. Sessini et al. [87] obtained a sandwich-type structure based on two external films of shape-memory Surlyn^®^ and an inner layer of electrospun PLA mat. 

Further studies based on PCL were performed by Yao et al. [88] based on the production of a PCL/PEO membrane by electrospinning, where PEO was added to improve the hydrophilicity of the membrane. PCL was irradiated with UV light to obtain cross-linked PCL in order to present shape-memory behavior: when deformed, the membrane, which presents a honeycomb-like structure, opens the structure, and the fibers are connected by cross-links. They studied the shape-memory effect for the PCL/PEO membrane in dry and wet conditions at 55 °C and it was observed that water (for the wet hybrid membrane) increased the recovery speed of the permanent shape, taking 12 s, in comparison with the dry hybrid membrane, which took 20 s.

PEO has been added several times in solution to adjust some properties. For example, Zhang et al. [89] used PEO to modify the viscosity of a Nafion^®^ solution and consequently to improve the electrospinnability in order to produce Nafion^®^ nanofibers with shape-memory properties stimulated by heat. In particular, DuPont Nafion^®^ PFSA is composed of a polytetrafluorethylene backbone and perfluorovinyl ether pendant side chains terminated by a sulfonate ionic group and is characterized by high chemical and thermal stability, acidity and electrical conductivity, and mechanical properties [90]. The fibers were subsequently annealed at different temperatures—140, 160, and 180 °C, respectively—to obtain a network, leading to an increase in the diameter of the fibers. Lastly, they studied SM properties by subjecting the nanofibers to three consecutive cycles. The fibers achieved excellent *R_f_* and *R_r_* values of 95–96% and 87–89%, respectively. Afterward, they investigated such properties by modifying the PEO content: 0.3, 0.5, and 0.7 wt.%, and *R_f_* and *R_r_* values of around 90% were obtained for all samples. An increasing trend in *R_f_* and a decreasing trend in *R_r_* were observed by increasing the PEO content [91]. 

Further studies focused on Nafion^®^ have been carried out by researchers to take advantage of its different, interesting properties. In 2016, Hu et al. [92] aimed to develop a composite with shape-memory behavior induced by electrically resistive Joule heating, which can be used when direct heating is not readily applicable [93]. The composite was obtained by grafting carbon nanotubes and Nafion/silica nanofibers onto carbon fiber mats, where carbon fiber was used as an electric conductor to trigger the electrically resistive Joule heat, CNT as a thermal conductor to transfer the heat to the SMP matrix, and Nafion/silica additionally worked as an insulator to slow down the heat exchange between the carbon fiber and the surrounding environment. They reported that the recovery of the permanent form was activated by applying a constant 4.0 V DC voltage and it took 150 s, achieving an *R_r_* of approximately 92%. 

**Figure 11:**
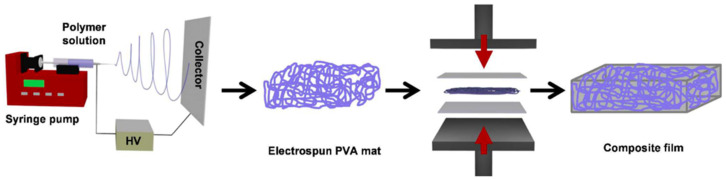
Representation of the production process of PEBA/PVA composite. Reprinted with permission from [94]. Copyright 2016 American Chemical Society.

Another polymer investigated by researchers is PVA. Shirole et al. [94] fabricated a shape-memory composite constituted by electrospun fibers of semicrystalline PVA as the switching element (by using T_g_ as transition temperature) and a thermoplastic polyether block amide elastomer (PEBA) as the matrix, by melt impregnation/compaction (Figure 11). The shape-memory performance revealed an *R_f_* of 66 ± 14% and an *R_r_* of 98 ± 2% at low strain (3–5%).

In 2020 Guan et al. [95] studied the variation of mechanical and shape-memory properties of PU nanofibers by changing nanofiber alignments: random, 0° (parallel to tensile direction), 45°, and 90°, respectively. Nanofibers with a 0° alignment degree showed excellent shape-memory properties with an *R_r_* higher than 93% and an *R_f_* higher than 90% and increased the yield stress, elastic modulus, tensile strength, and breaking strain by 22.6, 213.1, 35, and 39.5%, respectively, compared with randomly oriented nanofibers, due to the higher compactness and density of aligned nanofibers. Furthermore, it was observed that when the alignment angle decreases from 90° to 0°, mechanical properties are enhanced due to an increase in the volume fraction of nanofibers along the strain direction, which better bears the applied loading. 

Further research in order to optimize practical parameters was carried out by Banikazemi et al. [96] by fabricating shape-memory PCL-based PU fibers and studying the effect of solution concentration, type of solvent, feeding rate, needle-to-collector distance, and applied voltage. Beads-free fibers were obtained with a concentration of 5 *w/v*% of PU in 60/40 (DMF/chloroform) solvent. They observed that the fiber diameter decreased by increasing the needle-to-collector distance and the applied voltage; however, further increases led to an increase in the average diameter, while the diameter was also increased by increasing the feeding rate. The peculiar porous structure of the fibers where cavities act as weak points diminished the mechanical properties compared to those of bulk film, showing lower Young modulus, tensile strength, and elongational break as well as *R_f_* values. However, the electrospun scaffolds revealed *R_r_* values of 100%.

Nahavandizadeh et al. [97] fabricated in situ-polymerized shape-memory polyurethane hydroxyapatite (HA) scaffold and studied the effect of HA nanoparticles on electrospun fibers and shape-memory properties. The addition of HA led to a larger fiber diameter and a decrease in the degree of crystallinity, which derives from PCL soft segments, caused by the formation of hydrogen bonds between isocyanate molecules and OH- groups present on the nanoparticle surface, avoiding the introduction of PCL chains into the crystals. Furthermore, the increase in nanoparticle content limited the fast reformation of soft segments, leading to higher *R_f_*, and due to the small content of hard segments, the material showed *R_r_* values of 100%.

One of the most recent works in the scientific literature, focused on the development of a material with improved thermal and mechanical properties and with shape-memory properties, was reported in 2021 by Liu et al. [98]. They prepared a composite film by electrospinning, constituted by perylene-bisimide-functionalized graphene nanosheet (PBI–GN) obtained via π–π stacking between PBI, graphene, and PLA. The obtained material showed improved mechanical and thermal properties in terms of tensile strength and elongation at break and higher maximum thermal decomposition temperature and glass transition temperature. Additionally, the presence of PBI–GN improved the shape-memory capabilities of the material with respect to neat PLA (36.3% higher).

Another way to tailor the shape-memory behavior of SMPs directly involves some effects that are observed in many electrospun membranes—for example, the shrinkage that is caused by the stretched conformation of the macromolecular chains [99]. Other techniques are focused on the modification of the geometrical features of the fibers—for example, Sauter et al. [100] investigated the influence of the fiber diameter in the mechanical and shape-memory behavior of randomly electrospun polyetherurethane (PEU) meshes. Indeed, the mechanical and shape-memory properties strongly depend on the molecular orientation along with the orientation of the fiber, which is increased by the reduction in the fiber diameter from the micrometer to the nanometer range. Fibers with a diameter below 100 nm presented excellent shape recovery, while the shape fixity decreased with decreasing fiber diameter. 

In 2019, Leonés et al. [12] developed a PLA-based material and studied the modulation of the T_g_ of PLA through plasticization by blending PLA with oligomers of lactic acid (OLA), obtaining, for the first time, thermally-activated shape-memory electrospun fibers of neat and plasticized PLA for potential biomedical applications. In fact, OLA was revealed to be particularly compatible with PLA due to its chemical composition. Furthermore, OLA and lactic acid are the degradation products of PLA inside the body, which can be metabolized or eliminated through breath or urine [101].

**Figure 12:**
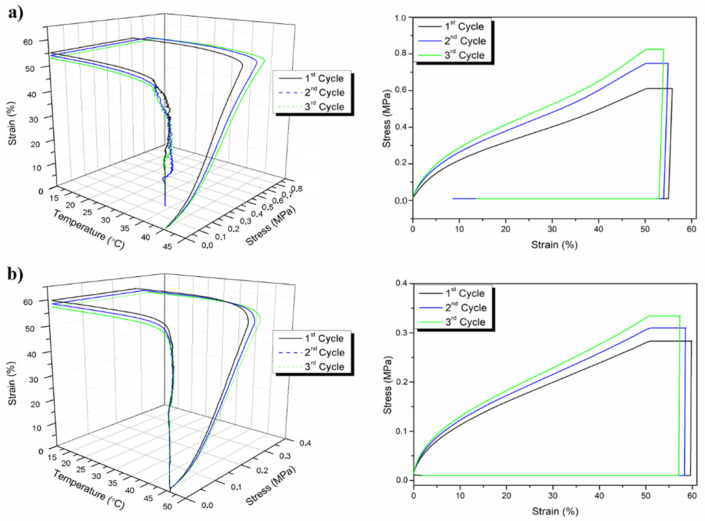
Thermo-mechanical shape-memory cycles of PLA-OLA (80:20) at (**a**) 40 °C and (**b**) 45 °C [12].

They observed a decrease in the average diameter of fibers by increasing the content of OLA, from 757 ± 193 nm for neat PLA to 768 ± 207 nm, 620 ± 121 nm, and 476 ± 80 nm for PLA-OLA 90:10, 80:20, and 70:30 ratios, respectively. Moreover, the system containing OLA presented a lower T_g_ than neat PLA (T_g_ = 60 °C), and the T_g_ decreased by increasing the OLA content from 50 °C (PLA-OLA: 90:10) to 35 °C (PLA-OLA: 80:20) and 20 °C (PLA-OLA: 70:30). The shape-memory properties were evaluated at 45 °C and 40 °C, temperatures close to that of the human body, with PLA-OLA ratios of 80:20 and 70:30, respectively. From the tests executed at 45 °C, with the formulation PLA-OLA 80:20, *R_f_* and *R_r_* values of 100% were achieved (*R_f_* > 95% after the first thermomechanical cycle), while at 40 °C it showed an *R_f_* = 98% and a slightly low value of *R_r_* due to the smaller temperature difference between the transition temperature and its T_g_. PLA-OLA 70:30 (with lower T_g_) presented better SM behavior at 40 °C than 45 °C. Indeed, at 45 °C it showed an *R_f_* = 90% and *R_r_* = 70%, whereas at 40 °C the *R_f_* and *R_r_* values were 92% and 75%, respectively. Among the studied materials, PLA-OLA with 20% of OLA was identified as the ideal material for biomedical applications, revealing the best mechanical properties at both testing temperatures, as reported in Figure 12.

Many researchers have been focusing on the study of structure, properties, and the optimization of parameters in order to obtain SMP fibers with high performance to be used for several applications. In particular, one of the areas in which SMP fibers have been widely used is the biomedical field, as reviewed in the following section.

## 3. Biomedical Applications

SMPs have been extensively used to develop scaffolds, implants, and biomedical devices due to their high biocompatibility and large shape deformation properties [102]. These purposes often require a quick shape recovery and sensitive response [14], and one of the challenges has been improving these properties without altering others. 

Potential biomedical applications for shape-memory electrospun nanofibers have gained increasing attention, in particular, in tissue engineering, drug delivery, wound dressing, and the development of biomedical devices and medical imaging.

### 3.1. Tissue Engineering

In 2013, Tseng et al. [103] developed a PU-based shape-changing scaffold for tissue engineering, especially to control the cell body orientation [104]. First, they deformed an initial random mesh (permanent form), obtaining an aligned mesh in a temporary shape, then human adipose-derived cells (ASCs) were cultured on the temporarily aligned scaffold. They observed that the cells preferentially oriented themselves according to the fiber orientation and that such preferential orientation was lost when the material recovered the permanent shape by heating at 37 °C; thus, the cells remained randomly orientated. However, in this case, the scaffold presented high shape-memory behavior with a shape fixing ratio of 99–100% and a shape recovery ratio of 94–96%.

**Figure 13:**
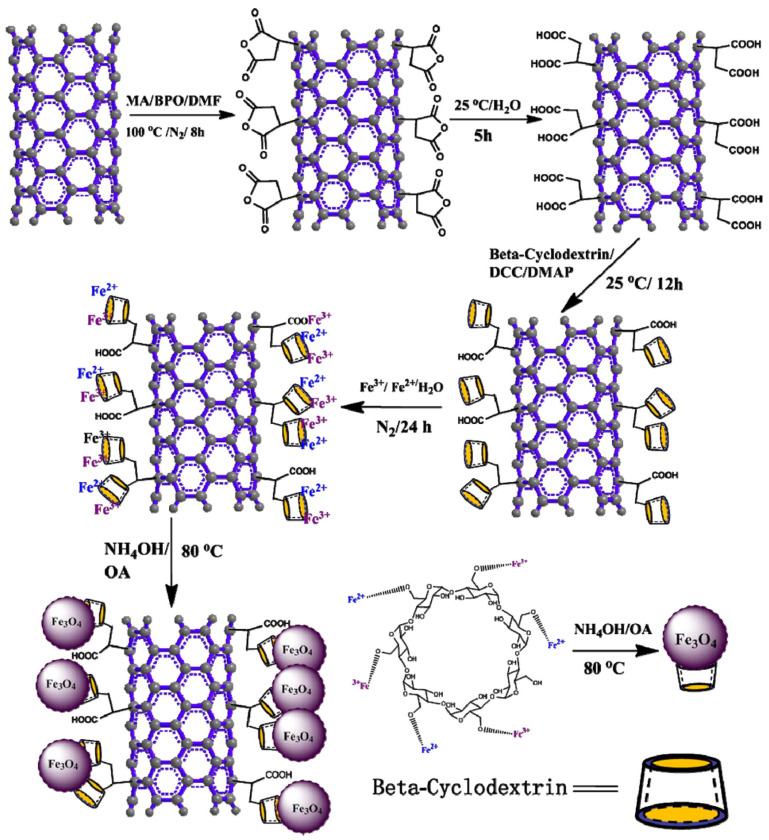
Synthesis of Fe_3_O_4_@CD-M nanoparticles. MWNTs were firstly functionalized by grafting maleic anhydride (MA) on their surface through a free radical reaction, subsequently modified by β-cyclodextrin (β-CD), and finally the formation of Fe_3_O_4_ nanoparticles by the co-precipitation of Fe^2+^ and Fe^3+^ using β-CD as depositional locus [105].

Gong et al. [105] fabricated polymer composite nanofibers by electrospinning, composed of a chemically cross-linked PCL (c-PCL) as the polymer matrix and multiwall carbon nanotubes coated with iron oxide (Fe_3_O_4_) nanoparticles (Figure 13) as reinforcement. The shape-memory effect can be triggered by hot water and by heat generated from Fe_3_O_4_ nanoparticles in an alternating magnetic field via hysteresis loss [106]. Actually, both stimuli induced shape recovery, but the recovery of the permanent form was reached faster when triggered by direct heat. This was probably caused by the high porosity that slowed the thermal conduction, recovering the initial shape more slowly when heated indirectly. Furthermore, the cytotoxicity of the material was evaluated through Alamar blue assay [107] by culturing osteoblast populations, revealing that the nanofibers, as well as their degradation product, were characterized by good biocompatibility, which is a fundamental property in tissue engineering.

Another interesting study was reported in 2014 by Bao et al. [108]. They prepared a thermoresponsive and biomimetic tissue engineering scaffold by electrospinning, constituted by poly(D,L-lactide-co-trimethylene carbonate) (PDLLA-co-TMC), obtained from copolymerization of the D,L-lactide (DLLA) monomer and the trimethylene carbonate (TMC) monomer. Following this procedure, they modulated the recovery temperature of the scaffold for applications in the human body and to regulate the mechanical and degradation properties by changing the molar ratio DLLA/TMC [109,110]. A decrease in the diameter of the fibers was observed: 1526 ± 120 nm, 1073 ± 201 nm, 790 ± 204 nm, and 682 ± 146 nm by varying the monomer ratio DLLA/TMC from 5:5 to 7:3, 8:2, and 9:1, respectively, also causing a variation of the T_g_ that can be exploited for biomedical applications. A DLLA/TMC ratio of 8:2 with a T_g_ of 36.7 °C and 9:1 with T_g_ = 44.2 °C were chosen for these purposes. In fact, molar ratios of 5:5 and 7:3 showed lower T_g_, and those materials with T_trans_ close to room temperature and human body temperature are subjected to automatically induced shape changes after implantation, while T_trans_ is slightly higher than body temperature, allowing better control of this behavior [111]. Shape-memory performances were evaluated and the fibers showed excellent shape-memory properties with an *R_r_* value above 94% and an *R_f_* value above 98%. Moreover, they investigated the bone formation by culturing rat calvarial osteoblast on the fibrous PDLLA-co-TMC scaffolds in vitro to verify the cytocompatibility as well as biomineralization-relevant outcomes, including alkaline phosphatase expression and mineral deposition.

With the aim of developing functional, environmentally-friendly materials, Wei et al. [112] produced an electrospun chitosan (CS)/PEO membrane with different mass ratios where CS acted as the hard domain and PEO as the soft domain. The material showed *R_f_* and *R_r_* values above 90% after five cycles.

Among the biomedical applications, the replacement of metal hardware for the treatment of bone fractures has been investigated, since the removal of such devices can cause microtrauma and can leave empty screw holes that can lead to a risk of refracture [113]. With the aim of developing a biomimetic nanofibrous scaffold/implant, in 2016, Bao et al. [114] incorporated nano-HAp into poly(D,L-lactide-co-trimethylene carbonate) (PLMC), which is a copolymer with shape-memory properties, by co-electrospinning. HAp was loaded at different contents—1, 2, and 3 wt.%—and the modulation of T_g_ was observed in the range between 43.5 and 51.3 °C by changing the HAp percentage. Furthermore, the composite material showed great SM properties, achieving *R_r_* and *R_f_* values above 99%. In this case, biological assay results confirmed that the incorporation of HAp in PLMC nanofibers considerably promoted osteoblast growth, alkaline phosphatase secretion, and mineral deposition in bone formation. 

Another biomedical issue concerns nerve regeneration. The peripheral nervous system (PNS) can be damaged as a consequence of different types of trauma, such as traffic accidents, natural disasters, and others. Generally, when the injuries are small, the nerve can regenerate itself, but when injuries are larger they need to be surgically treated with nerve grafts taken from other parts of the body [115]. Therefore, the production of flexible tubular scaffolds was studied to adopt new approaches [116] and, especially, the production of materials that present a prolonged and gradual recovery to mimic the regeneration process [117].

**Figure 14:**
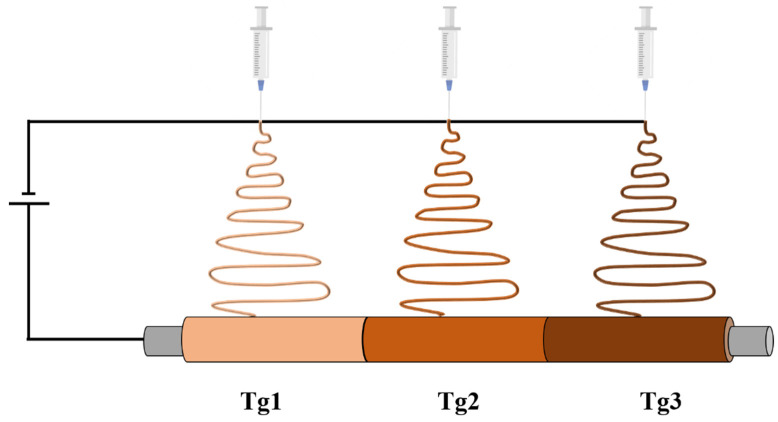
Representation of the fabrication of the trisegment SMP by electrospinning.

Chen et al. [118] developed a smart nerve conduit (SNC) with an automatic gradual lengthening of both proximal and distal nerve stumps through electrospinning. Such biodegradable SMP achieved gradual self-shrinkage in body water (36 °C). In order to obtain a prolonged recovery time, they fabricated a trisegment SMP with different glass transition temperatures for each segment (Figure 14).

PLLA was modified by substituting L-lactide with *rac*-lactide and then glycolide was incorporated into the network. Finally, poly(*rac*-lactide-*co*-glycolide) was obtained and the T_g_ was tuned by varying glycolide monomer constituents. The SM properties were evaluated: all networks showed an *R_f_* value of ~99% after ten thermomechanical cycles, whereas *R_r_* decreased from 90% to 50% during the first five cycles and the value stayed constant after the sixth cycle. This fact can be explained by a loss of physical crosslinks during the programming; however, the device will require only one activation. 

Many other researchers have studied the fabrication of SM scaffolds for tissue engineering applications. Kai et al. [119] developed an SM polyurethane copolymer constituted by organic PCL and inorganic PDMS at different ratios of PCL/PDMS (9:1, 8:2, 7:3) by electrospinning. The ratio variation led to different fiber diameters and thermal and mechanical properties. Moreover, the SM properties were evaluated, revealing an *R_r_* above 90% and an *R_f_* above 92% after seven cycles. Furthermore, the biocompatibility of the material was proven, since the copolymer supported the proliferation of human fetal osteoblastic (hFOB) cells in in vitro culture. The authors subsequently investigated the properties of the same material, also incorporating carbon black to fabricate electrically conductive nanofibers [120]. Indeed, it was observed that the electrospun conductive nanofibrous scaffolds enhanced the nerve regeneration process [121]. They also observed an increase in the fiber diameter from 399 ± 76 nm to 619 ± 138 nm by adding carbon black (since the presence of the filler increases the viscosity of the solution) and a decrease in the resistivity from 3.6 GΩ mm^−1^ to 1.8 kΩ mm^−1^. From the evaluation of SM properties, they noticed the almost complete independence of the SM behavior from the addition of the fillers, given that the *R_f_* and *R_r_* values showed a slight decrease, however, maintaining the shape recovery ratio above 90% and the shape fixity ratio above 82%, even after five cycles. In addition, the nanofibers showed good biocompatibility. PC12 cells were cultured on the scaffold and the nerve cells showed increased interactions. 

Mejia et al. [122] used gelatin, which is a natural polymer derived from the controlled hydrolysis of collagen with high biodegradability and biocompatibility [123], and SMPU, both combined with carbon nanotubes to construct a scaffold through electrospinning. Among the materials produced, SMPU with MWCNTs presented the best performance in terms of morphology, with nanofibers of an average diameter of 451 nm and greater mechanical properties with a tensile strength of 1.912 MPa and a high ratio of volume/surface.

In 2017, Marlettini et al. [124] developed a biodegradable and biocompatible scaffold starting from lactic acid, 1,3-propanediol, azelaic acid, and sebacic acid to obtain a PLLA-based triblock copolymer where the central block is poly(propylene azelate-co-propylene sebacate) (P(Paz60PSeb40)) random copolymer, which showed a thermally induced shape-memory effect in the physiological temperature range. In particular, P(Paz60PSeb40) acted as switching segments while PLLA blocks, with higher T_m_, acted as the physical network. In addition, instead of tuning the shape recovery temperature by varying the composition of copolymers, they used an alternative approach that consists of thermal annealing, where the sample is submitted to a controlled temperature for a limited time [125], which leads to an increased crystal phase perfection and consequently to a higher T_m_. After annealing at 15 °C, the material started the recovery at 25 °C and finalized it at 40 °C, while, after annealing at 30 °C, the recovery started at 35 °C and was completed at 55 °C. Both the samples exhibited very high values of *R_f_* and *R_r_*, that is 98% for the sample annealed at 15 °C and 99% for the one annealed at 30 °C. Moreover, the biocompatibility of the proposed scaffold was demonstrated by using NIH/3T3 fibroblast cells, showing high spread and cell proliferation.

SMPs for biomedical applications were also explored by Pandini et al. [126] by fabricating PCL-based fibrous mats through a combination of electrospinning and sol-gel reaction and, by varying the extent of the sol-gel reaction, two materials with a low and high crosslinking degree were obtained [127] (PCL-low and PCL-high, respectively). PCL-high, in particular, showed larger elongation–contraction effects and, as a result of biological investigation, higher cell proliferation and spreading. Since electrospinning permits the production of micro-porous structures similar to extracellular tissue where cells can be cultivated and proliferate [128], many studies were focused on the production of scaffolds, to develop implants for cardiovascular diseases that are functionally similar to blood vessels [129]. Montoya et al. [130] cultivated cardiac fibroblasts for more than 10 days of incubation in an SMPU membrane (Irogran) fabricated with two thicknesses, one with a thickness between 0.2 mm and 0.9 mm (sample 0) and the other between 0.9 mm and 1.0 mm (sample +1), and observed that sample +1 improved the growth and proliferation of the tissue cells in comparison to sample 0, since it presented less porosity and a drop in the permeability values due to the growth of the tissue cells.

Many other studies were carried out to find scaffolds that mimic the extracellular matrix (ECM), which permits cell motility during tissue repair [131], but also to control the cell migration in disease progression [132]. To mimic the dynamic behavior of ECM, Wang et al. [133] fabricated a synthetic 3D biomaterial scaffold, which in the presence of cells can modify the fiber alignment in cytocompatible conditions by triggering the shape change at temperatures from 30 to 37 °C when hydrated. The study of cellular response was conducted by choosing the fibrosarcoma cell line HT-1080 and the murine mesenchymal stem cell line C3H/10T1/2, as they present high metastatic cancer cell motility and classic fibroblastic mobility, respectively. They found that both kinds of cells could be switched between polarized motility along the direction of fiber alignment and non-polarized motility between aligned and unaligned morphologies. Furthermore, they observed that cell velocity can be increased by increasing the fiber alignment.

Another field where SMPs have been applied concerns the treatment of spinal cord injuries (SCI) that lead to the destruction of nervous tissue [134]. The regeneration of these tissues can be promoted by the transplantation of stem cells that supply new neurons [135], but, unfortunately, this technique is affected by low cell viability and uneven cell distribution. In order to solve these problems, in 2018, Wang et al. [136] developed an injectable cell delivery system based on hydrogel with shape-memory properties. Hydrogels are often employed for these purposes because of their biodegradability, easy processability, and minimally invasive and present structural properties similar to tissues and ECM [137]. The injectable composite was fabricated by embedding poly(D,L-lactic acid-*co*-trimethyl carbonate (P(DLLA-co-TMC)), fabricated by electrospinning, in gelatin-acrylated β-cyclodextrin (β-CD) polyethylene glycol-hydrogel (GCP-hydrogel), where the matrix created a local microenvironment for cell assembly: embryonic stem cells (ESCs) were used in this case and acted as a lubricant. The electrospun fibers showed an average diameter of 800 ± 200 nm, which remained unchanged when incorporated in the matrix. By observing the mechanical properties at 37 °C, it was noticed that nanomesh slightly reinforced the GPC-hydrogel. Furthermore, the shape-memory properties were evaluated by incubating the wrinkled hydrogel at 37 °C, the temperature at which it recovers its initial shape, and it was observed that the material recovers its original morphology in ~15 s with an *R_r_* > 95%. The authors discovered that the composite was capable of significantly improving the viability of ESCs and their differentiation towards motor neurons both in vitro and in vivo. 

The fabrication of materials to be used in the biomedical field and tissue engineering was studied also by Yao et al. [138] by incorporating Fe nanowire in an EVA matrix by electrospinning. The shape-memory behavior was obtained by a pre-treatment of the EVA sol with UV radiation to crosslink the material. The shape-memory behavior was investigated by applying two different stimuli: 55 °C hot water (contact stimuli) and light radiation (non-contact stimuli). The material recovers its initial shape in hot water after 6 s, whereas it took 100 s when irradiating, due to the fact that Fe nanowires are able to convert light energy to heat energy. 

Inverardi et al. [139] produced another scaffold based on crosslinked PCL, and instead of changing the microstructure by modifying the electrospinning set-up [140], they took advantage of its SM behavior in modulating the fiber alignment by fixing different temporary shapes with different microstructures. The material is able to fix and recover the temporary and permanent shapes even when high deformation was applied (100%). Moreover, neural stem cells derived from human iPSCs (induced Pluripotent Stem Cells) were seeded on both randomly and aligned fiber mats, showing cytocompatibility. It seems that in the randomly orientated mats, cell growth proceeded in all directions, while in the aligned fibrous mats, cell growth proceeded preferentially along with the fiber orientation. 

SMPs have also been studied to develop scaffolds to be used for treating vascular disorders that are generally caused by endothelial cell malfunctions by causing damage to the arterial wall [141]. The rapid formation of a confluent endothelial monolayer is paramount for vascular remodeling and regulating coagulation since the endothelial surface is constantly exposed to humoral factors, inflammatory mediators, and physical forces [142]. With the aim of producing artificial vascular grafts (tubular scaffolds), Zhao et al. [143] designed a shape-morphing scaffold capable of converting from a 2D planar shape (temporary shape) into a 3D tubular shape (permanent shape) at physiological temperature and where endothelial cells were previously seeded. The scaffold is obtained by the co-electrospinning of PCL and gelatin methacrylate (GelMA). PCL was chosen because it presents similar mechanical properties to blood vessels [144], while GelMA supports cell adhesion, proliferation, and migration [145]. In this case, the shape-morphing scaffold exhibited the characteristics required regarding structure, supporting cell attachment and growth.

Further studies regarding biomedical applications were carried out by Niiyama et al. [43] by developing a temperature-responsive nanofiber mesh of a PCL-based polyurethane with different ratios of soft segments (PCL diol) and hard segments (hexamethylene diisocyanate (HDI)/1,4-butanediol (BD)). They observed that mechanical, fiber processability and stability, T_m_, and SM properties can be changed by varying the PCL/HDI/BD ratio. The nanofibers exhibited great shape-memory performance, in particular PCL/HDI/BD 1:4:3. The nanofibers were placed in water at 60 °C and then stretched to different elongation degrees (200, 300, and 400%). Finally, they recovered their original shape when placed again in hot water (at 60 °C), showing high *R_r_* (>89%), even when deformed at the greatest elongation (400%). Moreover, human mesenchymal stem cells (hMSCs) were cultured on the meshes, showing alignment along with the fiber orientation, thereby demonstrating the biocompatible nature of the material.

**Figure 15:**
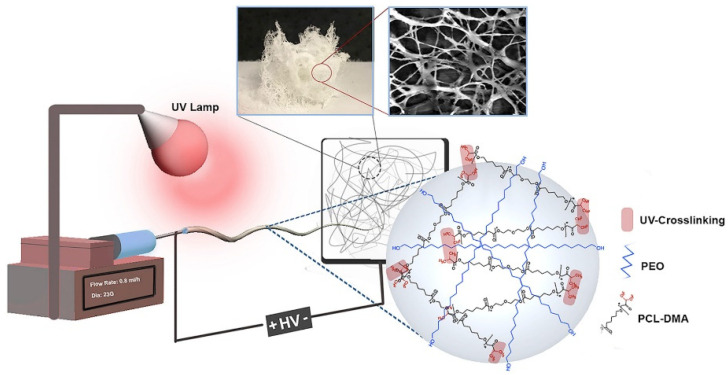
Representation of the sponge-like 3D scaffold [150].

Recently, multiple shape-memory polymers have attracted much attention, since they can be easily tailored by changing the transition temperatures without resorting to the synthesis of other polymers, and, besides, can be designed in several ways [146]. Multiple-SMPs are usually produced by blending two immiscible SM polymers characterized by two different transition temperatures [147], thus considering that the blending of two miscible polymers would lead to a homogeneous phase that would exhibit only one T_trans_ and dual-SM behavior. Therefore, with the aim of fabricating a material that showed triple-SM behavior constituted by miscible materials, Sabzi et al. [148] designed a triple-SMP consisting of PLA and PVAc, materials with two distinct T_g,_ through the co-electrospinning method, Additionally, graphene nanoplatelets were included to improve the SM behavior [149]. The material presented excellent triple-SM behavior, revealing high values of *R_r_* and *R_f_* and from cytotoxicity tests, high cell growth and the proliferation of the osteoblasts on the fibrous scaffolds containing graphene were observed. With this purpose, Zare et al. [150] developed a polycaprolactone dimethacrylate (PCLDMA) and PEO composite scaffold, in which PCLDMA acted as the switching segment (with a T_m_ close to body temperature) and PEO as the permanent segment, by self-assembly electrospinning and photo-crosslinking, which can be applied simultaneously [105,126], finally obtaining a highly porous, sponge-like scaffold (Figure 15).

Furthermore, gold nanoparticles (GNPs) of 10 nm were incorporated since they have been used in many biomedical applications, for photoablation, diagnostic imaging, as antioxidants, drug delivery vehicles, and for detecting cancer [151,152]. The sponge-like scaffold showed an average porosity of 93 ± 1.8%, providing high absorption abilities and good shape-memory capabilities, with *R_f_* and *R_r_* values of 97% and 88–89%, respectively. Cytotoxicity tests with NIH3T3 cells revealed more than 75% cell viability, hence proving the low cytotoxicity of the 3D sponges.

Since the electrospinning technique produces 2D woven no-woven scaffolds, in order to produce more suitable scaffolds for tissue engineering, the development of 3D scaffolds was studied to extend the potential applications, as they better mimic the structure of ECM and regulate cellular biological behavior (adhesion, differentiation, and matrix deposition) [153,154]. With a view of producing a 3D scaffold for cartilage regeneration, Chen et al. [155] adopted a different approach by processing 1D gelatin/poly(lactic-*co*-glycolic acid) (PLGA) electrospun fibers into inks ideal for 3D printing so as to obtain 3D scaffolds with a controlled shape and porous structure. Three-dimensional printing allows the fabrication of material with a precise form; however, the produced scaffolds presently lack fibrous surface structure, constituting a problem for the development of materials that mimic the ECM structure. Therefore, the combination of electrospinning and 3D printing should lead to the fabrication of a 3D scaffold with an appropriate, controlled shape and high porosity. The 3D scaffold presented showed good elasticity and water-induced shape-memory behavior, and, in addition, when combined with chondrocytes, achieved cartilage regeneration in vivo [155]. 

In 2020, Chen et al. [156] developed an SM tubular scaffold for blood vessel replacement composed of poly(lactide–glycolide–trimethylene carbonate) (PLGATMC) as an outer layer and an inner layer of poly(lactide-glycolide) (PLGA)/chitosan, where smooth muscle cells (SMCs) were cultured. PLGATMC was chosen for programming the deformation from a 2D planar, in which cells can be located, to a 3D tubular shape, assuming the blood vessel structure, whereas PLGA was selected for its biocompatibility, biodegradability, and mechanical properties [157]. Nevertheless, it presents low cell affinity because of its poor wettability and lack of cellular recognition sites that permit the biological signals. These cell recognition sites were provided by chitosan (CS) [158]. PLGA/CS@PLGATMC was fixed in the temporary shape at 20 °C, where cells were cultured, and then the 3D tubular permanent shape was recovered at 37 °C. It was reported that among the fabricated scaffolds with different PLGA/CS ratios, the one with a PLGA/CS ratio of 7:3 turned out to be the most suitable for tissue engineering purposes, displaying the best biocompatibility for SMCs. 

In order to fabricate materials for biomedical applications, polylactides have been widely used. However, as said before, their brittleness, low toughness, relatively poor retention, and recovery efficiency led the researchers to the production of polylactide-based shape-memory copolymers or blends with other polymers. In 2021, Wang et al. [159] modified PLLA by incorporating poly(3-hydroxybutyrate-co-3-hydroxyvalerate) (PHBV) to form ultrafine composite fibers by using the electrospinning technique. They identified the formulation of PLLA-PHBV (7:3) as the best formulation, since the presence of PHBV decreased the T_g_ and increased the Young modulus of the material. In particular, the material showed an *R_f_* (>98%) and an *R_r_* (>96%) compared to neat PLLA. Moreover, electrospun PLLA-PHBV composite fibers showed enhanced osteogenic-inducing ability in the mouse bone mesenchymal stem cells, which makes this fabricated scaffold suitable for bone tissue repair applications.

Further studies have been reported in 2021 in order to produce scaffolds and medical devices. For instance, Sarabiyan et al. [160] fabricated SMPU/graphene quantum dot nanoparticle (GQDs) nanofibers. The presence of PCL as the soft segment in the structure contributed to the shape-memory behavior of the material. The GQD NPs improved the mechanical properties of the nanofibers and more uniform nanofibers were obtained due to the electrical conductivity of GQD NPs that support the electrospinning process. Additionally, the presence of this kind of NPs enhanced the proliferation and compatibility of 3T3 fibroblast cells with the scaffold.

**Figure 16:**
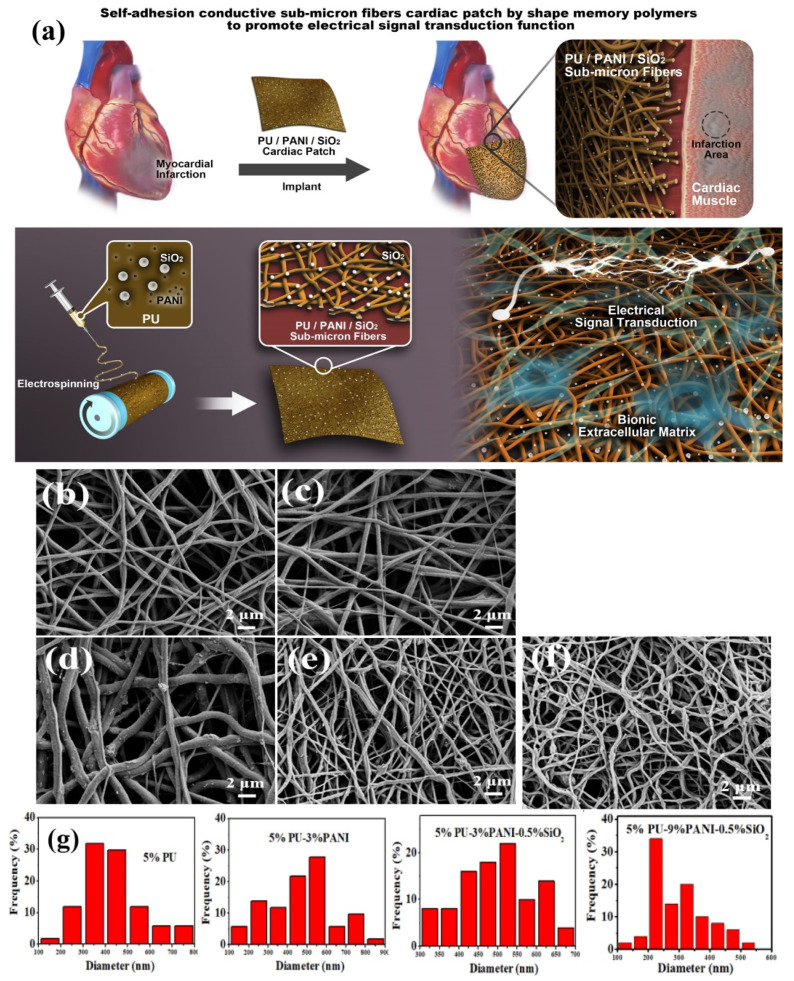
(**a**) Fabrication and self-adhesion of sub-micron fiber cardiac patch. SEM of (**b**) 5% PU, (**c**) 5% PU–3% PANI, and (**d**) 5% PU–3% PANI–0.5% SiO_2_, (**e**) 5% PU–6% PANI–0.5% SiO_2_, and (**f**) 5% PU–9% PANI–0.5% SiO_2_, and (**g**) fiber diameter distribution. Reprinted (adapted) with permission from [161]. Copyright 2021 American Chemical Society.

Another interesting work related to tissue engineering was carried out by Feng et al. [161], by fabricating a sub-micron fiber patch of polyurethane/polyaniline/silicon oxide (PU/PANI/SiO_2_) with a 3D porous structure and improved electrical signal transduction and self-adhesion, which simulated the myocardium ECM for myocardial infarction (MI) therapy. SMPU has been used for its capability to recover its original shape after a few seconds to minutes under specific conditions, while the presence of conjugated polymers such as PANI provides conductivity since electrical signals are fundamental for the heart’s activity, and, finally, the incorporation of SiO_2_ into biopolymers improves the bioactivity. The schematic procedure of the fabrication and self-adhesion of the sub-micron fibers cardiac patch is shown in Figure 16.

SMPUs were used also by Lv et al. [162] with the aim of fabricating light-responsive SMPs by the introduction of dopamine (DA) that was subsequently polymerized, obtaining a coating of polydopamine (PDA) onto the surface. PDA has good biocompatibility and presents the capability to produce heat when illuminated by NIR light, permitting the recovery of the permanent shape and observing the recovery of the initial shape by irradiating the material with a light intensity above 0.2 W cm^−2^ (taking 30 s under 0.6 W cm^−2^ and 18 s under 1.0 W cm^−2^).

With the aim of producing materials with shape-memory behavior, sometimes polymers without this property are modified in order to achieve the desired SM properties. For example, Liverani et al. [163] used PCL modified at chain-ends, producing a triethoxysilane-terminated poly(epsilon-caprolactone) (PCL-TES) loaded with bioactive glasses (BG), which are capable of binding the host tissue and generating a coating of hydroxycarbonate apatite. The SiO_2_ domains subsequently formed the reaction between the ends group, constituting the permanent crosslinking point. The chemical interactions between the PCL-TES chain ends and the silanol groups on the nanoparticle surface generated additional crosslinking points that permitted the recovery of the permanent shape, showing an *R_r_* and *R_f_* above 90%.

### 3.2. Drug Delivery 

Even if nanofibers have been widely used in the biomedical field for tissue engineering, drug delivery is another important field of application. Actually, the large surface areas and high porosity provide high loading capacity and encapsulation efficiency [164,165], thus allowing the fabrication of new scaffolds as drug carriers suitable for post-surgical adhesions and infections and postoperative local chemotherapy [166].

Another polymer that has been investigated is polydioxanone (PDX) since electrospun PDX presents similar mechanical properties to collagen and elastin [167] and has been widely used in drug/gene delivery. Moreover, PDX nanofibers have been studied as anti-inflammatory agents [168] in rotator cuff repair [169]. For instance, Kratz et al. [170] designed a diblock PCL-b-PDX nanofibrous scaffold that revealed values of *R_f_* and *R_r_* in the range of 92–98%. Besides that, they explored hydrolytic and enzymatic in vitro degradation.

Biocompatible SMPs have been widely produced, generally triggered by heating or by light. However, temperature activation is not always achievable or recommended. For this reason, some researchers have produced intelligent materials that can be triggered by biological activity: enzymatic triggering has been identified as one of the solutions, due to their biological activity and specific localization in the human body [171]. Additionally, SMPs triggered by enzymes can find applications in drug delivery, scaffolds for tissue regeneration, and platforms for stem cells. In 2019, Buffington et al. [172], designed a cytocompatible shape-memory polymer enzymatically triggered and constituted by PCL as a shape fixing component, which is vulnerable to enzymatic degradation, and Pellethane^®^ (5863-80A) as a shape-memory component that is enzymatically stable. The temporary shape was fixed sequentially by heating above the T_m_ of PCL, deforming the material, and, finally, by decreasing the temperature. In this step, PCL was maintained under compression and Pellethane^®^ under tension. Afterwards, the material was placed in a solution of lipase, which is an enzyme produced by both eukaryotes and prokaryotes [173], the hydrolyzed ester bonds leading to PCL degradation and the Pellethane^®^ recovered back its original shape. Through shape-memory properties evaluation, it was observed that such behavior was evident only when the highest lipase concentration was used (0.5 mg mL^−1^) within a 7 days test period. Indeed, lower concentrations of lipase degraded the PCL but no shape recovery was observed.

Lv et al. [174] designed an SMPU/HA composite (HA has been already used for bone tissue engineering given its large presence in bone ECM [47]), suitable as a drug carrier. Furthermore, they investigated the release of dexamethasone (DEX), a synthetic glucocorticoid that supports bone formation [175], which was previously loaded into the fibers. In this case, HA improved the degradation rate of the fibers: the weight loss after 10 weeks for the fibers with 1, 3, and 5 wt.% was 14.5, 17.1, and 19.1 wt.%, respectively, causing the simultaneous, controlled release of the drug that strongly depends on the polymer degradation. The material with 3 wt.% of HA showed the best SM performance with an *R_r_* > 97% and a recovery time of ~6 s. 

Another drug delivery system (DDSs) was developed by Yin et al. [176] by designing a multilayered electrospun structure (sandwich structural membrane) through sequential electrospinning and consisting of three layers of electrospun SMPU with a T_trans_ ≈ 42 °C. A natural antibacterial compound, berberine hydrochloride (BCH), was embedded in the middle layer, and the two outer hydrophobic layers were necessary to extend the drug release period. Such a design permitted the increase of the diffusion path of the drugs from the medium [177] to have better control over the drug’s delivery. The experiments revealed that 80 wt.% of BCH was released in 144 h and that the release rate was increased by stretching and fixing the temporary shape.

Bil et al. [178] fabricated another drug delivery system based on two types of SMPUs: PU-PLGA and PU-PLLA/PEG, which differed in soft segment composition by introducing D,L-lactide-co-glycolide diol (o-PLGA), poly-L-lactide diol (o-PLLA), or polyethylene glycol (o-PEG) in order to modulate the SM properties and decrease the T_trans_ to values close to body temperature. Both electrospun mats (PU-PLGA and PU-PLLA/PEG) showed an *R_f_* value above 90%, and *R_r_* values of 98% and 99% for PU-PLLA/PEG and PU-PLGA, respectively. In general, the PU-PLGA scaffold presented the best features in terms of mechanical properties while both materials are suitable as drug delivery systems for rapamycin (Rap), revealing a controlled release over 45 days with therapeutic dosage.

### 3.3. Wound Dressing 

**Figure 17:**
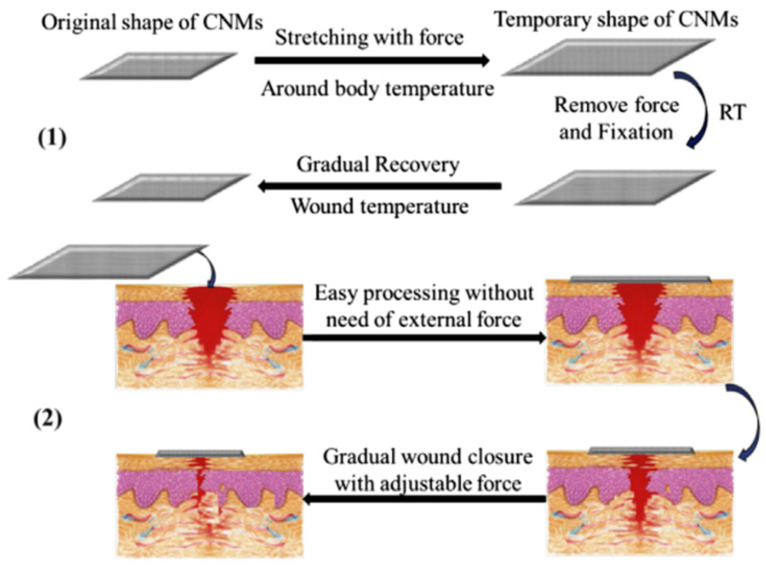
Schematic illustration of wound closure process by using CNMs [182].

Among the various biomedical applications, SMPs as wound dressings have also been studied. When the skin is damaged, a physiological process called wound healing takes place, which includes cell proliferation, differentiation, and migration, leading to tissue repair and regeneration [179,180]. Composite nanofibers, derived from synthetic and natural polymers, satisfy the conditions of wound healing in terms of structure and properties [28,181]. For this purpose, in 2015, Tan et al. [182] fabricated novel composite nanofibrous mats (CNMs) consisting of chitosan, gelatin, and SMPU by electrospinning. Chitosan and gelatin were incorporated to enhance the hydrophilicity and biological properties of the material while SMPU acted as a mechanical matrix. Then, to improve the antimicrobial properties, the material was treated with a silver nitrate (AgNO_3_) solution [183]. The SM properties were evaluated, obtaining an *R_r_* value above 95% after the first cycle and an *R_f_* value above 92% even after three cycles. Because of these SM properties, CNMs can be used for the wound closure process: the force accumulated by the pre-programming of CNMs is used to close the wound slowly, and later, this force is released when recovering the former shape (under body temperature) without applying any external force. Otherwise, immediate recovery could cause pain and nuisance (Figure 17). 

### 3.4. Biomedical Devices and Medical Imaging

Other approaches have been used in order to fabricate smart materials for practical applications such as medical imaging and for the fabrication of biomedical devices. In 2011, Zhang et al. [184] aimed to design a microfiber film based on PCL which, compared to the bulk SMPU film, achieved a quicker recovery of the permanent shape when heated in a water bath, obtaining an improvement of such properties without changing the chemical composition of the starting material. The shape recovery of the microfiber film took ~¼ time of that needed for the bulk film to reach an *R_r_* value from 10% to 90%. This behavior was attributed to the higher surface area of the fibers, leading to an easier diffusion of water and, consequently, a faster heating/cooling of the material. 

Other studies have been directed to the design of dye-containing polymers. In particular, dyes that absorb and emit light in the near-IR spectrum have also found a place in medical imaging applications in order to replace other, more invasive techniques such as x-ray imaging. Near-infrared fluorescence (NIRF) (700–1000 nm) imaging has been investigated as an alternative since the main tissue absorbing components present minimal absorption in this range: oxy- and deoxyhemoglobin (λ_max_ < 600 nm) and water (λ_max_ > 1150 nm). In 2014, Torbati et al. [185] incorporated indocyanine green (ICG), which is a dye that presents an adsorption band around 700 nm and an emission band around 800 nm [186], into a PVAc matrix to produce a fibrous web by electrospinning, finding an optimum dye concentration (0.0125 mg mL^−1^) that gave the highest emission intensities. Moreover, they discovered that the fiber mats showed higher fluorescence intensity and higher uniform dye incorporation with respect to the cast films. Furthermore, fibers contract upon heating or in contact with water due to the SM properties of PVAc. Such behavior can be used for applications such as feeding tubes or catheters.

Zhang et al. [187] developed a core/shell composite fiber by coaxial electrospinning, in which epoxy is the reinforced core layer that provides good mechanical properties and PCL is used as a shell layer, which improves the biocompatibility of the composite; indeed, a cytotoxicity test was carried out. SM properties were also evaluated: the material can recover its initial shape when heating at 70 °C, the complete shape recovery took 6.2 s, and the R_r_ was about 100%. From the cytotoxicity test, performed with a CCK-8 assay, it was observed that the cell viability of the PCL/epoxy membrane was more than 80% after 4 days of cultivation, proving the non-toxicity of the material.

Kuang et al. [188] used PU-based architecture and 3,4-dihydroxy-1-butene (DHB) as a chain extender with a pendant allyl group suitable for the formation of crosslinks and for controlling the melting temperature of the material, consequently altering the SM properties. Their aim was to fabricate a biodegradable, crosslinkable, and electrospinnable thermoplastic PU with two-way reversible SM behavior. The material showed good shape fixing and recovering behavior and, additionally, they observed that PCL_3k_-TPUs underwent enzymatic degradation in a two-step way that depends on their crystalline structure and their crosslinking degree, demonstrating the biodegradability of the material, which makes it potentially suitable for biomedical applications. 

Peng et al. [189] fabricated PLA nanofibers reinforced with hyperbranched PLA-modified cellulose nanocrystals (H-PLA-CNCs) by electrospinning, potentially suitable as a membrane. PLA has been widely studied as a biocompatible and biodegradable material, used particularly in the biomedical sector [190]. However, it presents poor mechanical properties and hydrophilicity. Thus, to overcome these problems, CNCs were added as reinforcement, presenting an abundance of hydroxyl groups on the surface that led to easy incorporation and dispersion into the matrix [191]. The SM performance was evaluated and compared to that of neat PLA, which showed an *R_f_* value of 90% and an *R_r_* of 56%, and it was observed that by adding 1, 3, and 5 wt.% of H-PLA-CNCs, the *R_r_* increased from 67 to 77 and 93%, respectively. On the other hand, by adding 7 wt.% of H-PLA-CNCs, the *R_r_* decreased to a value of 42.9%. The presence of hydrogen bonds between H-PLA-CNCs and PLA allowed the recovery of the initial shape under the traction of the hydrogen bond force, but with the highest amount of H-PLA-CNCs, the membrane was subjected to an irreversible deformation, leading to a lower *R_r_* value.

Hsieh et al. [192] developed electrospun copolymer nanofibers with potential applications as medical shrinkable tubing and wire, based on PCL and PDMS with different PCL/PDMS ratios, where PCL acted as the switching segment and determined the crystallinity of the copolymer nanofibers. The fabricated copolymers performed SM properties with a response of 10 s to recover its original shape and *R_r_* and *R_f_* values around 98% and 100%, respectively, triggered by a temperature around 37 °C, which is the melting temperature revealed from all the produced materials for all compositions. 

Many studies were focused on the study of structure, properties, and the optimization of parameters in order to obtain fibers with high performance and to be used for several applications; in particular, in the biomedical field. However, the SM properties are suitable for applications in many other fields.

## 4. Other Applications

Even though shape-memory nanofibers have found applications in tissue engineering, wound dressing, and drug delivery, some researchers have been focusing their attention on the improvement/modulation of the properties of these materials for further potential uses.

One of the fields investigated is robotics, in which SM fibers can find applications as microswitches, microactuators, and micromanipulators (actuators and sensors). However, the polymeric material, in order to be potentially used, should respond to an external stimulus such as a current or electric field with a subsequent strain. Some of them, in reverse, show the capability to transform mechanical movement into an electrical response [193,194]. In 2010, McDowell et al. [194] fabricated an electroactive polyferrocenylsilane (PFS) functionalized with pendant alkoxysilane groups, subsequently hydrolytically gelled by adding *p*-toluene sulfonic acid hydrate (PTSA·H_2_O) that also increased the conductivity of the electrospinning solutions, leading to a production of fibers with a low number of structural defects. These materials demonstrated strain-induced deformation on electroactuation at 1.5–2.0 V anodic potential, producing a reversible 20% longitudinal strain, revealing, therefore, the capability to convert an electrical signal to a mechanical response, whereas the application of 2.0 V cathodic potential led to the recovery of the initial shape.

Among the various alternative applications, additional studies have been carried out by investigating the antibacterial activity to use them as active antibacterial nanomaterials [195]. In 2011, Zhuo et al. [196] fabricated SMPU core-shell nanofibers by coaxial electrospinning, constituted by a core of polycaprolactone-based SMPU (CLSMPU) and a shell of pyridine-containing polyurethane (PySMPU) and obtaining both the core-shell and bead-on-string structure. The shape-memory performances of the core-shell nanofibers were studied and the material revealed an *R_f_* value above 95%, significantly improved compared to that of pure CLSMPU nanofibers, which present a lower *R_f_* value (~80%) [34], while the *R_r_* showed values above 90%. The CLSMPU-PySMPU nanofiber revealed outstanding antibacterial activity by showing no growth of *S. aureus* and *K. pneumoniae* bacteria beneath the mat, while this growth was observed with the pure CLSMPU mat. 

Additionally, since the electrospinning technique permits the fabrication of porous materials, the application of shape-memory electrospun nanofibers as a membrane has been also studied. Ahn et al. [197] developed SMPU membranes with two-way shape-memory behavior in order to modify the size of the nanopores. The material was subjected to one cycle of SM behavior and then the material changed from the permanent to the temporary shape without the “programming” step. With a bias load (constant stress) applied, the material expanded upon cooling and then shrunk by heating at temperatures higher than T_trans_, recovering the initial shape when the retracting force exceeded the stress applied. Upon cooling, the average pore size increased from 154 to 444 nm, thus allowing the separation of substances with different sizes only by changing the temperature. The modulation of the pore dimensions by changing the temperature has been used also for the development of intelligent clothing material.

Chung et al. [198] aimed to fabricate electrospun nanowebs (ESWs) of SMPU with different hard segment content and PCL molecular weight (M_n_). The specimen with higher PCL M_n_ (4000 g mol^−1^) with higher hard segment content (33 wt.%), which showed an R_f_ value of 86% and an *R_r_* value of 85%, was chosen to fabricate the ESW, revealing good moisture and air permeability due to the high porosity when kept in its expanded state below the transition temperature. 

In 2013, Rodriguez et al. [199] fabricated a soft anisotropic shape-memory elastomeric composite (A-SME-C) that can be used in the automotive and aerospace industries in laminated composite elastomers. They aimed to produce a material that could mimic the skin membrane of a bat wing, which presents anisotropic properties: the wing membrane skin shows high stiffness and strength parallel to the skeleton and high extensibility parallel to the wing’s trailing edge. This anisotropy is caused by the disposition of the fibers of both collagen and elastin [200]. A poly(vinyl acetate) (PVAc) mat was incorporated as reinforcement into a Sylgard-184 silicone (Sylgard) elastomeric matrix. Moreover, the elastic modulus (E_y_), strain-to-failure (ε_f_), and yield stress (σ_y_) were evaluated according to the fiber orientation angle χ: 0° (longitudinal), 22.5, 45, 67.5, and 90° (transverse), observing E_y_ average values of 48.8 MPa and 4.0 MPa, ε_f_ of 198% and 351%, and σ_y_ of 4.6 MPa and 0.9 MPa for longitudinal and transversal loading, respectively. From the SM performance, a decrease in *R_f_* was observed by decreasing the orientation angle χ: from 95.6% for 0° to 86.2% for transversal oriented fibers (90°), while *R_r_* maintained high values (around 99%) independently from the fiber orientation. 

Zhang et al. [201] developed Nafion^®^-based quintuple shape-memory membranes that can find applications in robotics and aerospace engineering, and also as a catalyst. Electrospun Nafion^®^ nanofiber membranes (ENNMs) presented higher specific surface area, higher aspect ratio, and higher pore diameter compared with the bulk films, and these features were employed to improve the catalytic activity [202], which was also enhanced by adjusting the shape of the material by varying the temperature. The quintuple-SM behavior was achieved by establishing four T_trans_: 160, 125, 90, and 55 °C, and the Nafion membrane revealed *R_f_* and *R_r_* values around 80%, while the *R_r_* to recover the initial and permanent shape was above 70%. 

In 2014, the same authors reported another research developing an SM nanocomposite constituted by a Nafion® polymeric matrix with shape-memory behavior and PAN-based carbonization nanofiber, which makes the material sensitive to electrical stimulus [203]. The PAN nanofibers were firstly subjected to stabilization and carbonization processes [204] to obtain a network and finally integrated into the matrix. The average fiber diameter decreased from 618 ± 67 to 327 ± 38 nm, caused by the chemical changes to which the material was subjected during the consecutive procedures. Additionally, by increasing the applied voltage from 20 to 25 and 30 kV, the average diameter of the PAN-based carbonization fibers also increased from 343 ± 49 to 483 ± 48 nm, leading to a decrease in conductivity. The recovery of the permanent shape was triggered by applying a 14 V constant electrical current, taking only 5 s, and the material revealed excellent SM properties with an R_r_ value of approximately 100%. 

In 2015, Nejad et al. [205] carried out the production of a composite material consisting of PVAc and PCL by dual-electrospinning and manifesting both self-healing (SH) and SM behavior upon thermal activation in order to obtain an SH coating or a potential packaging material. Such material manifested dual-SM behavior in the dry state and triple-SM behavior in the wet state. 

Fan et al. [206] fabricated a hierarchical porous carbon nanofibrous (CNF) membrane by electrospinning and doping with different contents of SiO_2_ (5, 10, and 20 wt.%)_,_ which can find applications in the adsorption of proteins. The SiO_2_@CNF exhibited improved mechanical properties, high water permeability, and enhanced protein absorption capacity for bull serum albumin. The introduction of SiO_2_ nanoparticles significantly improved the mechanical properties: in fact, by increasing the nanoparticle content, the stiffness of the membranes was decreased, as was revealed by the decrease in the elastic modulus. The shape-memory capabilities were observed by previously bending the sample with 20 wt.% of SiO_2_ to a radius of <100 μm, without observing any rupture, followed by a subsequent recovery of the initial shape. The enhanced mechanical properties were attributed to the “plasticizer effect” of the embedded nanoparticles.

In 2018, Del Sorbo et al. [207] carried out the production of light, non-woven mats by electrospinning a solution of PVP where graphene was previously dispersed, observing, for the first time, the non-monotonous effects of the addition of graphene. Graphene is considered an “environment-friendly material” that exhibits high-flame retarding, and for this reason, is widely used in engineering [208]. They observed that the graphene concentration affected, in a non-monotonous way, the electrospinning process: at low concentrations, greater fiber stretching and lower fiber diameter were observed, whereas, at higher concentrations, the diameter of the fibers increased. The reason for this trend was attributed to the instability of the electrospun jet, particularly evident at low graphene concentrations, which seemed to be strongly connected to the acoustic properties of the material that changed in a non-monotonous manner.

Zhang et al. [209] fabricated a conductive shape-memory microfiber membrane suitable as a sensor and actuator, consisting of SM PLA microfibers obtained by electrospinning and a polypyrrole (PPy) coating obtained through chemical vapor polymerization by using FeCl_3_ as a catalyst. PPy, in this case, substituted the conductive particles that are generally added in order to provide electrical actuation; however, these fillers can present limitations related to non-uniform dispersion, thus the authors adopted another approach that consisted of the fabrication of core-shell fibers to obtain an almost uniform temperature within the material when electrically activated. The membrane showed a maximum conductivity of 0.5 S cm^−1^ and the recovery of the permanent shape was achieved in 2 s by applying a voltage of 30 V. Furthermore, it was observed that above 40 V, the heating damaged the membrane, while no SM effect was observed by applying a voltage below 15 V. 

Moreover, Khalili et al. [210] aimed to develop a material suitable as a sensor, in particular a strain sensor, which converts the mechanical compression into electrical signals and that, among various applications, can be used to monitor the motion of the human body by evaluating the strain-dependent changes in device performance [211]. Hence, they fabricated a nanofibrous mat composed of TPU and PLA at different percentages, coated with single-walled carbon nanotubes (SWCNT) to provide electrical conductivity to the surface of the fibers. The fibers could be stretched to 100% strain without influencing the mechanical properties, and the material that showed the best performance in terms of maximum mechanical and electrical recovery was TPU/PLA 25:75 wt.%. Additionally, the fibers exhibited shrinkage in length by increasing the PLA content and the TPU/PLA 25:75 wt.% underwent a maximum length reduction of 50%. 

In 2019, Zhang et al. [212] developed electrospun fiber meshes that were able to display a reversible stimuli-responsive pore size change under stress-free conditions, for potential application as a membrane for air filtration, given the high porosity, large range of pore sizes, and high permeability for gases [213]. Semi-crystalline linear PCL, which was further chemically cross-linked, was utilized to produce electrospun fibrous meshes with reversible bidirectional shape-memory polymer actuation. The polymers that perform this behavior can be actuated under stress-free conditions between two different shapes [214]. The actuation performance was carried out by running heating–cooling cycles between 60 °C (T_m_ of PCL) and 10 °C, with the specimen programmed with a strain of 100% and 300%. It was observed that when heating above 60 °C, the material was subjected to contraction, and to elongation when cooling. Furthermore, the reversible strain was measured [214] for PCL, obtaining a value of 6 ± 1% when a programming strain of 100% was applied, and an increased value of 22 ± 1% when applying a programming strain of 300%. Additionally, a change in the pore size was observed, from an average value of 10.5 ± 0.5 at 60 °C to 11.8 ± 0.6 µm at 10 °C. 

The interest in the fabrication of devices suitable for health services has widely increased in the last years, for example in the development of systems to purify different proteins that can be used as therapeutic agents [215] and that can be separated through high-performance liquid chromatography. Therefore, a lot of researchers have realized the fabrication of high-performance chromatographic media to substitute the conventional particle-based ion-exchange chromatographic (IEX) media [216], which is considered the reference technique since it separates charge variants by differential interactions on charge support. This number increases with the molecular weight of the sample; however, these media are limited by long retention times, low throughput, and high-pressure drops due to the small pores and the limited accessibility of adsorption sites [217]. 

To overcome these limitations, in 2019, Fu et al. [218] aimed to design nanofiber-based protein adsorption media with a high specific surface area and continuous structure. In particular, a highly phosphorylated nanofibrous aerogel (PNFAs) was designed through subsequent electrospinning, cryogenic induced phase separation regulation, and in situ phosphorylation modification. PNFAs exhibited high underwater superelasticity with a large initial modulus and high compression fatigue resistance (~0% plastic deformation even after 1000 cycles), even in ultra-cold liquid nitrogen, and good shape-memory behavior, and in this way could provide good column-packing properties and high-pressure resistance. Furthermore, the media showed a high protein adsorption capability of 3.3 ± 10^3^ mg g^−1^, a large liquid flux of 1.5 ± 10^4^ L m^−2^ h^−1^, and high selectivity to lysozyme from egg white. 

Ambient energy harvesting has become the object of interest of many researchers in order to produce mobile devices for capturing, accumulating, and storing energy for later use [219,220]. In 2019, Xiong et al. [221] designed tunable, microarchitectured shape-memory triboelectric nanogenerators (mSM-TENG). Triboelectric nanogenerators (TENGs), in particular, are devices able to convert ambient mechanical energy into electricity and have found applications in energy harvesting [222]. SMPU was used to produce three types of microstructural mats: microfibers (MFs), microspheres (MSs), and microspheres-nanofibers (MSNFs), with tunable surface roughness, self-restoring capabilities, and SM behavior triggered by temperature, among the produced mats. In particular, MSs were chosen to design a water temperature sensor on the deformed mat that showed increased surface roughness during recovery, which could increase the electrical output. 

Moreover, Guan et al. [223], in 2020, aimed to develop multifunctional nanofibers composed of lead zirconate titanate (PZT) and SMPU with piezoelectric and shape-memory behavior for energy harvesting. Piezoelectric materials can convert mechanical vibration into electrical energy, and these materials are widely used for energy harvesting [224]. These particles are often coated with coupling agents to improve their dispersion in the polymer matrix. In this case, PZT particles were added in different contents and modified by silane-coupling agents, resulting in a better dispersion. Therefore, PZT/SMPU harvested energy from sinusoidal vibrations and the sample with 80% of PZT produced voltages of 120.3 mV (peak-to-peak). Furthermore, SM performance was evaluated and all the PZT/SMPU nanofibers exhibited *R_r_* values above 94% after the first cycle and *R_f_* values higher than 98% in all three cycles. 

Yoon et al. [225] fabricated an SMP-TSE (transparent and stretchable electrode) composite constituted by a continuous silver nanofiber (AgNF) network with optical and electrical properties, where electrical conductivity can be increased with flexibility, and crosslinked polycyclooctene (PCO) as a stretchable substrate with shape-memory behavior, showing *R_f_* values above 96% and *R_r_* values above 97%, which revealed switchable optical transparency and potential applications as skin-like electronic devices.

Shape-memory polymers are widely used in the field of aeronautics, intelligent robots, and electronics. However, the traditional fabrication methods, e.g., injection molding and liquid molding, produced materials that could crack when they are twisted, limiting their applications. In order to overcome these problems, in 2021, Liu et al. [226] developed a novel device that can be used in an electrical circuit on graphene nanosheets, using sisal cellulose microcrystals as reinforcements, polyamine-functionalized perylene bisimide derivative (APBI) as a surfactant, and PVA. Additionally, they deposited Ag nanowires by spin-coating to improve the conductivity of the PVA films. The film, prepared by electrospinning, was made into an origami crane with the wing opened at 90° at room temperature (permanent shape), then heated at 90 °C for 2 min in order to modify the shape of the film. Subsequently, the temporary shape with the wing opened at 0° was fixed by cooling, and finally, the former shape was almost totally recovered (88°) by heating at 86 °C. 

More recently, concerns regarding the energy crisis related to global electricity consumption led researchers to develop new materials in order to find new solutions for these issues. In this regard, for instance, Feng et al. [227] fabricated a smart textile through coaxial electrospinning with moisture and thermal management capabilities which is capable of regulating the skin temperature and therefore reducing the usage of air conditioning. Elastic PU elastomer, which possesses soft-hard segments that permit the recovery of the permanent shape, was used as a “shell” and the PEG as a “core”. The T_trans_ is determined by the melting temperature of PEG (60 °C), then the fixation of the temporary shape and the recovery of the permanent shape can be triggered by the phase transition of PEG. The membrane presented excellent shape-memory behavior, which also allows the adjustment of the pore size of the membrane, to transport the moisture in excess and keep the skin dry.

Izraylit et al. [228] developed a soft actuator that has found applications in soft robotics by blending a multi-block copolymer containing PLLA and PCL segments with oligo(d-lactide) (ODLA) and studied the influence of the sample hierarchical structure on the shape-memory actuation capabilities.

**Figure 18:**
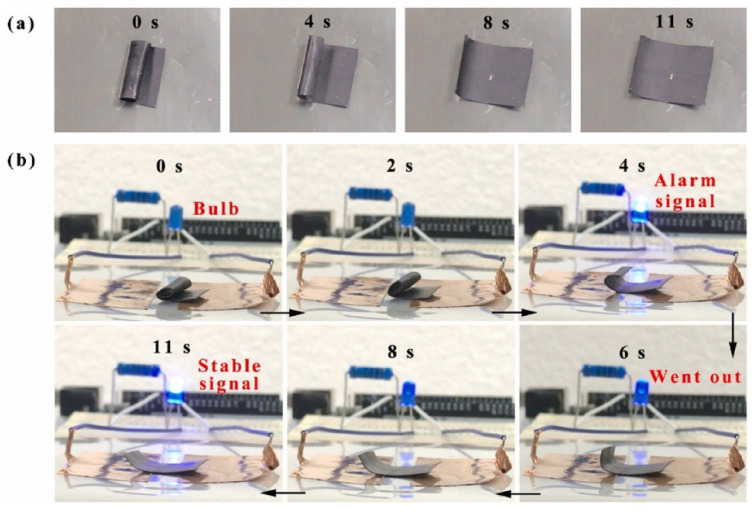
(**a**) Shape recovery of rolled-up paper. (**b**) Shape-memory behavior of the SMPU/MXene and its potential use as a fire alarm sensor [229].

One of the last works was carried out by Zhang et al. [229] by fabricating a fire alarm sensor via electrospinning and vacuum-assisted filtering and constituted by SMPU and MXene, which is a new generation of 2D materials constituted by a few layers of metal carbides or nitrides, finding applications in electrocatalysis and photocatalysis [230]. They found that the recovery of the permanent shape can be triggered by electricity with a voltage of 5 V due to the Joule heat produced by the MXene coating or by heating above the T_g_ of SMPU within 10 s. The mechanism of shape recovery and its potential use as a stimuli-responsive sensor is shown in Figure 18. They fixed the rolled-up shape as the temporary shape, maintained below the T_g_ of SMPU, and the circuit remained disconnected. When heated above T_g_, the paper recovered the unrolled shape, connecting the two copper tapes and forming a closed circuit. Furthermore, the SMPU/MXene showed self-extinguishing performance and fire retardancy with respect to SMPU paper, due to the barrier effect of Mxene, with a peak of heat release rate (pHRR) and total heat rate (THR) of 66.0 and 49.8%, respectively.

## 5. Conclusions and Future Perspectives

The progress in the optimization of the parameters, the architecture, the shape-memory behavior, the triggering methods, and the various applications of electrospun shape-memory nanofibers have been summarized in this review. 

Tremendous progress has been made since the first research was reported in terms of the study of the electrospinning parameters, which is a suitable, versatile, and cost-effective method that allows a simple way to fabricate continuous polymeric fibers. These electrospun fibers can show shape-memory properties, adjusting their architecture in order to obtain materials with one-way, two-way, and multiple SM behavior. Moreover, the fabrication of electrospun nanocomposites has also been studied and optimized in order to improve their properties and thus extend their field of applications and the triggering stimulus, such as temperature, pH, water, and electric fields as the most important.

Different application fields have been reviewed, with particular attention to the use of shape-memory electrospun nanofibers in the biomedical field, taking advantage of their capability to mimic the structure of the extracellular matrix as scaffolds in tissue engineering, tissue repair, wound dressing, and drug delivery. The cell behavior after culturing on the electrospun fibrous scaffold and the shape-memory behavior of the materials have been widely evaluated in vitro, however, it is still a challenge to study these features on cells in vivo. Furthermore, shape-memory electrospun nanofibers, particularly those triggered by the magnetic or electric fields, have recently extended their applications due to novel fabrication techniques in the aerospace field and in robotics, as actuators, sensors, for energy harvesting, etc.

Finally, despite the significant and recent progress in the fabrication of shape-memory polymers obtained by electrospinning, further studies are required and several challenges still need to be addressed to optimize the working parameters. Moreover, deeper efforts on the material design are required to exploit the advantages of polymers with multiple shape-memory behaviors with great potential for specific advanced applications in the biomedicine field, robotics, and electronics. 

## Data Availability

Not applicable.

## Abbreviations

A-SME-C	anisotropic shape-memory elastomeric composites
AgNF	silver nanofibers
AgNO_3_	silver nitrate
APBI	polyamine-functionalized perylene bisimide derivative
ASCs	adipose-derived cells
APS	γ-aminopropyltriethoxysilane
ASPU	end-capped polyurethane oligomer
BCH	berberine hydrochloride
BD	1,4-butanediol
BG	bioactive glasses
β-CD	β-cyclodextrin
C3H/10T1/2	murine mesenchymal stem cell line
CLSMPU	polycaprolactone based SMPU
CNF	carbon nanofibrous
CNMs	composite nanofibrous mats
CS	chitosan
1D	one-dimensional
2D	two-dimensional
3D	three-dimensional
DA	dopamine
DDSs	drug delivery system
DEX	dexamethasone
DHB	3,4-dihydroxy-1-butene
DLLA	D,L-lactide
DMF	dimethylformamide
DGEBA	diglycidyl ether of bisphenol A
E_y_	Young modulus
EAP	electroactive
ECM	extracellular matrix
ENNMs	electrospun Nafion^®^ nanofiber membranes
EP	epoxy
ESCs	embryonic stem cells
ESWs	electrospun nanowebs
EVA	ethylene-vinyl acetate
FeCl_3_	iron chloride
Fe_3_O_4_	iron oxide
FTIR	Fourier Transform Infrared Spectroscopy
GCP-hydrogel	polyethylene glycol-hydrogel
GelMA	gelatin methacrylate
GNPs	gold nanoparticles
GO	graphene oxide
GQDs	graphene quantum dot nanoparticles
H-PLA-CNCs	hyperbranched PLA-modified cellulose
HA	hydroxyapatite
HDI	hexamethylene diisocyanate
Hfob	human fetal osteoblastic
hMSCs	human mesenchymal stem cells
HT-1080	fibrosarcoma cell line
HUVECs	human umbilical vein endothelial cells
iPSCs	induced Pluripotent Stem Cells
IEX	ion-exchange chromatographic
M_n_	molecular weight
MFs	microfibers
MI	myocardial infarction
MSs	microspheres
mSM-TENG	microarchitectured shape-memory triboelectric nanogenerators
MSNFs	microspheres-nanofibers
MWNTs	multi-walled carbon nanotubes
NIH3T3 cells	mouse embryonic fibroblasts
NIRF	Near infrared fluorescence
OCL	oligo(ε-caprolactone)
ODLA	oligo(d-lactide)
OLA	oligomers of lactic acid
OPDO	oligo(p-dioxanone)
P(Paz60PSeb40)	poly(propylene azelate-co-propylene sebacate)
PAN	polyacrylonitrile
PANI	polyaniline
PBI–GN	perylene-bisimide-functionalized graphene nanosheet
PCL	poly(ε-caprolactone)
PCL-diol	polycaprolactone-diol
PCLDMA	polycaprolactone dimethacrylate
PCL-TES	triethoxysilane-terminated poly(epsilon-caprolactone)
PCO	polycyclooctene
PDA	polydopamine
PDC	copolyetheresterurethane
PDS	polydioxanone
PDLLA-co-TMC	poly(D,L-lactide-co-trimethylene carbonate)
PDMS	poly(dimethylsiloxane)
PDX	polydioxanone
PEBA	polyether block amide elastomer
PEG	poly(ethylene glycol)
PEO	polyethylene oxide
PES	polyethersulfone
PEU	polyetherurethane
PFS	polyferrocenylsilane
PHBV	poly(3-hydroxybutyrate-co-3-hydroxyvalerate)
pHRR	peak of heat release rate
PLGA	poly(lactide-glycolide)
PLGATMC	poly(lactide–glycolide–trimethylene carbonate
PLA	poly(lactid acid)
PLLA	poly(L-lactic acid)
PLMC	poly(D,L-lactide-co-trimethylene carbonate)
PMMA	poly(methyl methacrylate)
PNFAs	phosphorylated nanofibrous aerogels
PNS	peripheral nervous system
PPDL	poly(x-pentadecalactone)
PPy	polypyrrole
PS	polystyrene
PTSA·H_2_O	*p*-Toluene sulfonic acid hydrate
PU	polyurethanes
PVA	poly(vinyl alcohol)
PVAc	poly(vinyl acetate)
PVP	polyvinylpyrrolidone
PySMPU	pyridine containing polyurethane
PZT	zirconate titanate
*R_f_*	strain fixity ratio
*R_r_*	strain recovery ratio
Rap	rapamycin
SH	self-healing
SiO_2_	silicon oxide
SM	shape memory
SMCs	smooth muscle cells
SMPs	shape-memory polymers
SMP-TSE	transparent and stretchable electrode
SNC	smart nerve conduit
SWCNT	single-walled carbon nanotubes
T_g_	glass transition temperature
T_m_	melting temperature
T_trans_	transition temperature
TSPCs	triple-shape polymeric composites
TDI	2,4,2,6-Toluene diisocyante
TENGs	triboeletric nanogenerators
THR	total heat rate
TMC	trimethylene carbonate
TPS	thermoplastic starch
TPU	thermoplastic polyurethane
TSMPs	triple shape-memory polymer
ε_f_	strain-to-failure
σ_y_	yield stress
